# Development of a patient-reported outcome questionnaire for aplastic anemia and paroxysmal nocturnal hemoglobinuria (PRO-AA/PNH)

**DOI:** 10.1186/s13023-020-01532-3

**Published:** 2020-09-17

**Authors:** Kimmo Weisshaar, Hannah Ewald, Jörg Halter, Sabine Gerull, Sandra Schönfeld, Yuliya Senft, Maria Martinez, Anne Leuppi-Taegtmeyer, Nina Khanna, Birgit Maier, Antonio Risitano, Regis Peffault de Latour, Andre Tichelli, Jakob Passweg, Beatrice Drexler

**Affiliations:** 1 Division of Hematology, University Hospital Basel, 4031 Basel, Switzerland; 2 University Medical Library, University of Basel, 4051 Basel, Switzerland; 3 Basel Institute for Clinical Epidemiology and Biostatistics, Department of Clinical Research, University Hospital Basel, 4031 Basel, Switzerland; 4 Department of Diagnostic Hematology, University of Basel, 4051 Basel, Switzerland; 5 Department of Clinical Pharmacology and Toxicology, University and University Hospital Basel, 4051 Basel, Switzerland; 6 Infectious Diseases and Hospital Epidemiology, University Hospital Basel, 4031 Basel, Switzerland; 7 Department of Psychosomatic Medicine, University Hospital Basel, 4031 Basel, Switzerland; 8 Hematology, Department of Clinical Medicine and Surgery, Federico II University of Naples, Naples, Italy; 9 Severe Aplastic Anemia Working Party of the European Group for Blood and Marrow Transplantation, Leiden, Netherlands; 10 French Reference Center for Aplastic Anemia and Paroxysmal Nocturnal Hemoglobinuria, Saint Louis Hospital and University Paris Diderot, Paris, France

**Keywords:** Aplastic anemia, Paroxysmal nocturnal hemoglobinuria, Patient-reported outcome, Quality of life, Symptom

## Abstract

**Background:**

The introduction of new therapy modalities has significantly improved the outcome of aplastic anemia (AA) and paroxysmal nocturnal hemoglobinuria (PNH) patients. However, relatively little is known about the exact disease burden of AA/PNH since standardized assessments of symptoms including health-related quality of life (HRQoL) are frequently missing or inadequately designed for this rare patient group. We aimed to develop AA/PNH-specific questionnaires for self-reporting of symptoms, which could be included in electronic platforms for data collection and patient care.

**Methods:**

By scoping review, we extracted any reported symptoms in AA/PNH and their prevalence from the literature (Phase I). Consensus rounds with patients and medical experts were conducted to identify core symptoms reported in the literature and to add missing items (Phase II). Ultimately, AA/PNH-specific patient-reported outcome (PRO) questionnaires including the selected measures were designed (Phase III).

**Results:**

AA symptoms from 62 and PNH symptoms from 45 observational studies were extracted from the literature. Twenty-four patients and seven medical experts identified 11 core symptoms including HRQoL issues after three consensus rounds. Significant differences in the symptom ranking of patients versus medical experts could be observed. Therefore, patient- as well as expert-centered PRO questionnaires in AA and PNH were created following the concepts of validated instruments.

**Conclusion:**

The development of symptom self-reporting questionnaires for AA and PNH was feasible and the disease-specific PRO questionnaires can now be validated within a web-based workflow in a subsequent feasibility study.

## Background

Aplastic anemia (AA) and paroxysmal nocturnal hemoglobinuria (PNH) are two very rare hematologic diseases [1], which can overlap or occur independently. While considerable progress has been made in the understanding of the pathophysiology and treatment of AA/PNH in the last decades, relatively little is known about the exact disease burden on physical, mental and social health. With the implementation of the international PNH registry, clinical symptomatology and health-related quality of life (HRQoL) issues have been addressed systematically for the first time in PNH [2], asking for patient-reported symptoms at enrollment and documenting HRQoL by using the European Organization for Research and Treatment of Cancer Quality of Life Questionnaire (EORTC-QLQ)-C30 [3] and the Functional Assessment of Chronic Illness Therapy Fatigue Instrument (FACIT-Fatigue) [4]. However, the latter two instruments were primarily developed for cancer patients. In AA a comparable registry does not exist, hence symptoms and HRQoL are mainly published as side observations within case reports, observational studies and interventional trials. This lack of standardized symptom assessment in AA/PNH has recently led a German expert group to develop the first disease-specific HRQoL questionnaire for AA and/or PNH [5, 6]. This marks a key step for improving data collection in this rare disease group. Nonetheless, the current standard method for recording symptoms includes paper forms filled out by medical staff instead of patients directly. This could lead to underreporting as symptoms are undetected by clinicians up to half the time [7]. In consequence, this approach has been questioned to be efficient, and concerns have been raised that it reflects only partially the real patient experience [8]. As an alternative method, direct reporting of symptoms by patients as ‘patient-reported outcomes’ (PRO) has been increasingly advocated to improve data collection, quality of care and patient outcome [9]. In particular, electronic systems offer accessible and feasible options for recording PRO (ePRO) on a regular basis by patients. Combined with a feedback-system to physicians this has shown to improve HRQoL and adherence to therapy, reduce admissions to the emergency department or hospital, thus leading to a better overall survival in cancer patients [10, 11]. Based on these considerations and since systematic PRO collection has not been performed in these rare diseases so far, we saw a high potential of ePRO in AA and/or PNH. To achieve this objective, we designed the following study in three phases: First, we conducted a scoping review to collect any reported symptoms of patients with AA and/or PNH in the literature. Second, we aimed to identify core symptoms from the literature review as well as missing items by consensus rounds with medical experts and patients. In this context, we were particularly interested in measures indicating significant health deterioration and life-threat. The results of the scoping review and consensus rounds built the basis for the third phase, in which we developed concise disease-specific PRO questionnaires for AA/PNH patients, which could be incorporated into an electronic platform for data collection and patient care in the future.

## Methods

### Phase I (scoping review)

We carried out a scoping review to provide a synthesis of patient-reported symptoms in AA/PNH. Scoping reviews yield quantified results about the knowledge available on a particular topic, differing from systematic reviews as they focus on a broader research question and less on the quality of evidence [12].

### Eligibility criteria

We included patients of all ages diagnosed with either acquired AA and/or PNH. Patients with inherited bone marrow failure have been excluded. Studies reporting clinical symptoms related to AA/PNH and adverse events of commonly used AA/PNH therapies (e.g. ciclosporin, anti-thymocyte globulin, hematopoietic stem cell transplantation, eculizumab) were eligible. We did not include signs found by clinical examination (e.g. thrombosis, splenomegaly) or results of laboratory-based investigations (e.g. cytopenia, hemolysis parameters). Randomized controlled trials (RCT), observational studies as well as case reports/series were the basis for the review. We excluded animal-only studies, abstract-only publications, editorials, reviews, comments, letters, correspondences, conference abstracts and expert opinions. English and German literature published between 1980 and the 05. May 2020 was reviewed.

### Identification of relevant literature

A comprehensive scoping search on Medline and Embase via Ovid was conducted to determine the feasibility of the search. We developed the search strategy in consultation with a medical information specialist (HE) experienced in systematic reviews. Our search strategy included terms around the population and concept of PRO (Supplementary Table 1). We used text words and subject headings. Compared to Medline, Embase offered a subject heading for the term ‘paroxysmal nocturnal hemoglobinuria’ and retrieved more hits, hence we decided to run a full search on Embase via Ovid only (Supplementary Table 2).

### Data collection and analysis

#### Study selection

Two reviewers (BD, KW) identified inclusion and exclusion criteria for the title/abstract- and the full-text screening (Supplementary Tables 3 and 4). The title/abstract screening and the screening of all potentially relevant full texts were done in duplicate (BD and KW) in batches of 100 and 53 references, respectively. Any disagreements regarding eligibility were resolved by joint review and discussion. When the researchers reached a consensus of at least 90%, one reviewer (KW) continued the screening process of the remaining references alone.

#### Data extraction

One reviewer (KW) extracted study characteristics of the included studies categorized by disease (AA, PNH, AA-PNH) and study type (observational and RTCs, case series and reports). Symptoms related to AA and/or PNH as well as adverse events associated with AA/PNH treatment were recorded. Patient-reported symptoms related to comorbidities were not extracted. In case of similar meaning or synonymous usage, the terms were merged (e.g. hemoglobinuria and dark urine, limb pain and myalgia, dizziness and vertigo). The term ‘bleeding’ was further classified according to the WHO grading: minor bleeding (WHO grade 1–2) and major bleeding (WHO grade 3–4) [13]. Thereafter, all captured symptoms were put in order according to their frequency.

Since self-assessment of HRQoL in AA/PNH was limited in the literature, we supplemented the data extraction with qualitative research results obtained by Groth et al. [5, 6]. In this recent project, AA/PNH patients and physicians rated questions of the EORTC QLQ-C30 questionnaire according to their importance in AA/PNH. The highly relevant rated items were included in our data extraction.

### Phase II (consensus rounds)

#### Eligibility criteria

AA and/or PNH patients as well as nurses and physicians experienced in the care of AA/PNH were eligible for the consensus rounds. Patients and medical experts required basic language skills in German or English.

#### Recruitment

Patients were either recruited at the University Hospital Basel, during an AA/PNH patient symposium in Ulm, Germany or by contact with patient advocacy groups in Germany (Aplastische Anämie & PNH e. V. and Stiftung lichterzellen). Participating physicians and nurses were mostly from the Hematology Division of the University Hospital Basel, Switzerland. In addition, we consulted the chairperson and secretary of the Severe AA Working Party (SAAWP) of the European Bone Marrow Transplantation Society (EBMT) for their expertise.

#### Consensus rounds using the Delphi-technique

The literature review yielded a large number of symptoms and a selection process was required to identify the core symptoms in AA/PNH. For this purpose, we applied the modified Delphi-method, a well-established and previously validated system of developing consensus [14, 15]. The technique works as a group communication process that aims to achieve a convergence of opinions on a specific matter after multiple rounds of evaluation. Three rounds have been conducted, which in previous studies have shown to be sufficient for reaching a consensus [16].

For the first two rounds, the questionnaires were sent out to the participants either by mail or e-mail. The panel was asked to review the questionnaire within 4 weeks and to rank the importance of the respective symptom on a Likert-type scale ranging from one to four (Importance scale: 1 = not at all, 2 = a little, 3 = quite a bit, 4 = very). Additionally, open feedback on missing items could be given. The questionnaires were anonymous and identified by a dedicated number for the second consensus round.

After the first and second round, the mean rating of each item was calculated. The symptom questions were additionally modified and supplemented according to the open feedback.

In the third round, patients and experts were asked to select exact 11 items for the inclusion in the final questionnaire. The questionnaire limit of 11 items was based on previous literature showing a high practicability of the usage of 10 to 12 symptom items in PRO questionnaires, which could be completed quickly by the average patient (max. 20 min) [17].

### Phase III (design of a disease-specific PRO questionnaire)

The core symptoms identified by the previous consensus rounds formed the basis for further development of a corresponding patient-friendly questionnaire. For this we relied on the questionnaire design used in a comparable patient population (i.e. clinical cancer research and common PRO instruments, e.g. EORTC QLQ-C30, FACIT, PRO-CTCAE) as these have demonstrated validity, reliability and sensitivity [18–20].

## Results

### Phase I (scoping review)

#### Results of the literature search and study characteristics

**Fig. 1:**
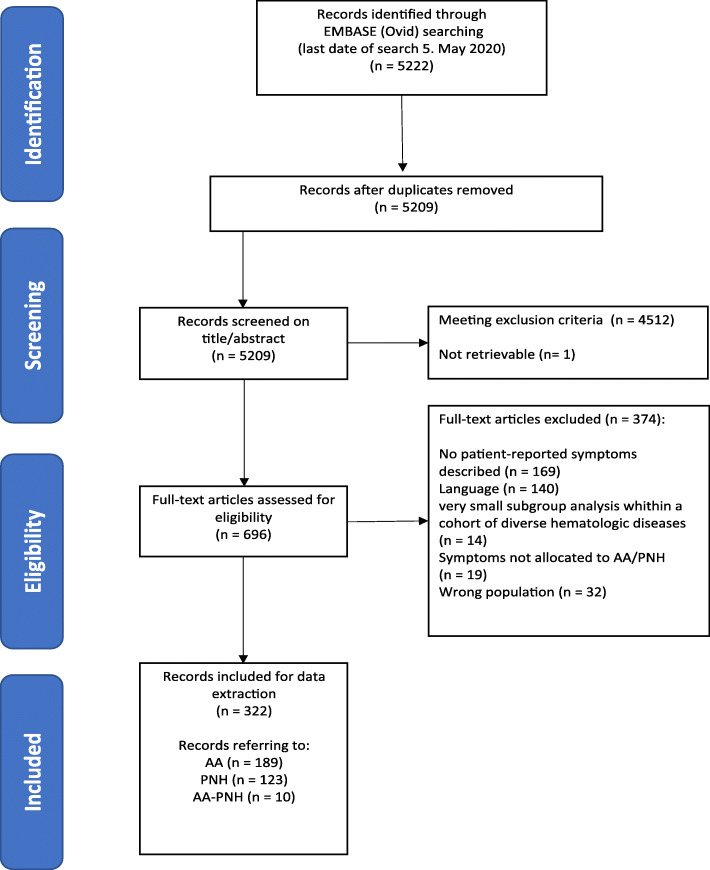
Study flow diagram for the selection of studies

The Embase search yielded 5222 records (last date of search: 05. May 2020) On the basis of the title and abstracts 4512 records were excluded. One record could not be retrieved by the university medical library and the publisher (Pakistan Pediatric Journal) did not respond to an inquiry. A total of 696 potentially eligible records were screened in full text. Of these, 322 publications (*n* = 189 for AA, *n* = 123 for PNH, *n* = 10 for AA-PNH) met our inclusion criteria and thus were used for data extraction (Fig. 1).

In AA, we found one RCT, 62 observational studies and 126 case reports/series. The records for PNH included three RCT, 45 observational studies and 75 case reports/series. The AA-PNH syndrome was primarily addressed by four observational studies and six case reports/series (Supplementary Table 5–10).

#### Data synthesis and analysis

The included studies (RCTs, observational studies) comprised 15′599 individuals (AA *n* = 6851, PNH *n* = 7521, AA-PNH *n* = 1227). Case reports/series covered further 328 patients (AA *n* = 201, PNH *n* = 120, AA-PNH *n =* 7). Partial overlap of the cohorts was found in eight studies of the PNH international registry or national PNH registries [2, 21–27], as well as in three studies by Hillmen et al. [28–30] with the SHEPHERD [31] and one of the TRIUMPH trials [32].

**Fig. 2:**
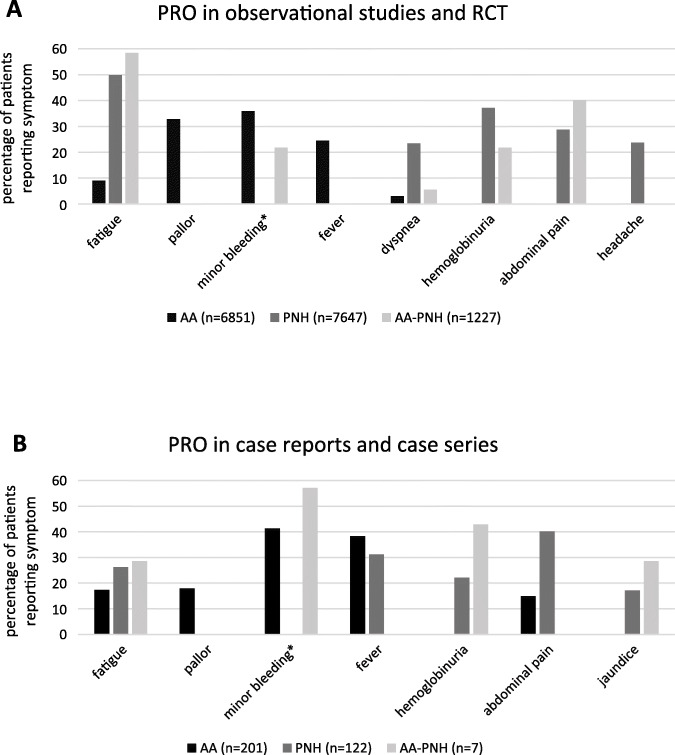
Patient-reported outcomes (PRO) in AA, PNH and AA-PNH in the literature. **a** Comparison of the frequency distribution of symptoms in observational studies and RCT between AA, PNH and AA-PNH. **b** Comparison of the frequency distribution of symptoms in case reports/series between AA, PNH and AA-PNH. Displayed are only the five most frequently reported symptoms of each disease. *WHO bleeding grade 1–2

A total of 62 different symptoms could be extracted for AA, 46 symptoms for PNH and 22 for AA-PNH. The majority of symptoms in AA in the observational studies were related to cytopenia (minor bleeding 35.9% > pallor 32.8% > fever 24.5% > fatigue 9.0% > and dyspnea 3.0%). In PNH patients, symptoms were most often associated to hemolysis (fatigue 49.8% > hemoglobinuria 37.2% > abdominal pain 28.7% > headache 23.7% > and dyspnea 23.4%). In the AA-PNH overlap patients, symptoms distinctive of both AA and PNH were reported (fatigue 58.4% > abdominal pain 40.2% > hemoglobinuria 22% > minor bleeding 21.8% > dyspnea 5.5% and others < 5%). The most commonly reported symptoms in RCT and observational studies were comparable to the symptoms presented in case reports/series (Fig. 2 and Supplementary Table 11 and 12).

### Phase II (consensus rounds)

The extracted PRO from the literature review were incorporated into a questionnaire with closed symptom questions for AA and PNH separately, both also covering symptoms of the overlap AA-PNH syndrome. Where possible, we merged symptoms into groups (e.g. nausea, vomiting, diarrhea and constipation to gastrointestinal problems) to ease the answering process. The first AA questionnaire comprised 19 questions on clinical symptoms and 8 questions on HRQoL, whereas PNH questions focused on 22 symptoms and 8 HRQoL issues (Supplementary Table 13).

#### 1st Delphi round

The panel of the first Delphi-round consisted of 31 participants (patients: 24, hematologists: 5, hematology nurses: 2). Thirteen patients (mean age 54.4 y; f:m = 6:7) evaluated the AA questionnaire and eight (mean age 39.3 y; f:m = 4:4) the PNH questionnaire. Three patients (mean age 36.0 y; f:m = 2:1) completed both questionnaires since they were affected by either condition (Supplementary Table 14), likewise, the seven medical experts returned both questionnaires.

All items of the AA questionnaire were answered by 98% and in the PNH questionnaire by 97% of the participants. Symptoms and HRQoL questions were rated according to the predefined Likert Scale (1 = not at all, 2 = a little, 3 = quite a bit, 4 = very) and little open feedback was given. In the AA questionnaire, the following symptoms were stated as missing by the patients: limb cramps and spasms (*n* = 2), tremor and ataxia (*n* = 1), hand sensory dysfunction (*n =* 1). One expert suggested to additionally cover the quality of family time and availability to children. Within the PNH questionnaire, no additional item was requested. Patients and experts suggested that related items should be grouped. With few exception (Supplementary Table 15) symptoms covered by the AA as well the PNH questionnaire were rated similarly by the two disease groups according to the mean Likert score.

Compared to the patients’ mean rating, the experts rated vital parameters remarkably higher (Supplementary Table 17).

#### 2nd Delphi round

As a result of the first round the AA questionnaire was condensed to 26 and the PNH questionnaire to 27 items for the second consensus round. The overall response rate in the second round was 84.4%, of which all items of both questionnaires were answered by 97% of the participants. Four patients (PNH *n* = 3 and AA-PNH *n* = 1) and one expert were lost to follow up. Again, most symptoms in AA and PNH were assessed similar by patients. In contrast, experts rated disease specific symptoms, such as increased bleeding tendency in AA and hemoglobinuria in PNH, significantly higher than patients. Contrary to the idea of the Delphi method no convergence of the mean patient and expert ratings could be observed when comparing the first with the second consensus round. (Table 1, see also Supplementary Table 19).

**Table 1:** Comparison of the mean rating (Likert scale) of questionnaire items from the second consensus round between patients and medical experts. Importance rating: 1 = not at all, 2 = a little, 3 = quite a bit, 4 = very. Number of participants evaluating the questionnaire: AA patients *n* = 13, PNH patients *n* = 5, AA-PNH patients *n* = 2, experts *n* = 6

PRO-AA/PNH Questionnaire items	Mean rating	
	AA Patients; experts	PNH Patients; experts
1. In general, do or did you feel tired?	3.3; 3.7	3.4; 3.8
2. Did you experience shortness of breath?	2.9; 3.7	2.6; 3.8
3. Do you have an increased bleeding tendency?	2.7; 3.8	1.9; 3.3
4. Were you limited in doing either your work or other daily as well as leisure time activities?	3.1; 3.3	2.7; 3.0
5. Did you have difficulties in concentrating on things?	2.4; 2.7	2.9; 2.5
6. Do you have any trouble doing strenuous and/or long-lasting activities? (e.g. carrying a heavy bag, taking long walks)	3.1; 3.2	2.6; 2.7
7. Was your mood impaired? (feeling depressed, being worried, feeling tense and others)	2.5; 2.7	3.1; 2.5
8. Did you have fever? (from 38.1 °C at least 2 times or once ≥38.3 °C)	1.9; 3.8	2.1; 3.7
9. Did you record a high blood pressure? (upper value > 140 mmHg, lower value > 90 mmHg)	1.9; 2.8	1.4; 2.7
10. Was your sleep impaired? (difficulties in falling asleep, staying asleep or waking up)	2.7; 2.2	2.9; 2.2
11. Have you been in pain?	2.2; 3.2	2.7; 3.8
12. Have you noticed any changes in hair, skin and/or mucous membranes?	2.7; 2.7	2.1; 2.0
13. Did you feel dizzy/lightheaded/unsteady?	2.4; 2.8	2.4; 2.5
14. Did you record a too low or to high pulse? (< 60 beats/minute or > 90 beats/minute)	2.5; 2.8	1.6; 2.2
15. Did you experience one or more changes in your sensory perception?	2.0; 2.3	1.6; 2.5
16. Did you suffer from muscle cramps/spasms?	2.8; 2.5	N/A
17. Did you experience tremor ^a^ and/or ataxia ^b^? (^a^ uncontrolled shaking movements of the whole or parts of the body; ^b^ lack of coordination of muscle movements)	1.7; 2.5	N/A
18. Was the time with your family and/or your availability to your children impaired?	2.5; 2.7	N/A
19. Do you need to stay in bed or a chair during the day?	1.9; 2.3	1.7; 2.2
20. Did you have digestive/gastrointestinal problems?	2.2; 2.3	2.1; 3.0
21. Did you have a cough?	1.7; 2.5	2.0; 2.5
22. Did you have swelling/edema of your limbs?	1.9; 2.3	1.9; 2.3
23. Did you lose or gain weight unintentionally?	2.0; 2.2	2.4; 2.3
24. Did you experience palpitations ^c^? (^c^ unpleasant sensation of irregular and/or forceful beating of the heart)	1.7; 1.8	2.3; 2.3
25. Did your skin feel itchy?	1.5; 2.0	1.6; 1.8
26. Have you noticed a dark discoloration of the urine?	N/A	2.9; 3.8
27. Have you noticed a yellowish discoloration of your ‘white of the eye’?	N/A	1.6; 3.2
28. Men only: Do you suffer from erectile dysfunction ^d^? (^d^ inability to achieve or to maintain an erection during sexual activity)	N/A	1.4; 3.0
29. Did you have difficulties in swallowing things?	N/A	1.6; 2.7
30. When was your last IV infusion of eculizumab?	N/A	1.9; 3.3

*N/A* not applicable

#### 3rd Delphi round

Due to divergent opinions on the main PROs between patients and experts in the first two rounds, we decided to draft separate PRO questionnaires within the final third Delphi round: *expert*-centered questionnaires for AA and PNH assessing life-threatening events and complications (“red flags”) and *patient*-centered questionnaires for AA and PNH focusing less on physical constraints indicating complications and more on HRQoL.

Among the experts there was a strong consensus to include the highest-rated symptoms from the previous rounds into the final questionnaire, but the panel (JRP, AT, JH, SG, BD) decided on several modification: i.e. exclusion of GvHD associated symptoms, merging similar content and addition of less known symptoms (palpitation, concentration problems) (see supplementary Table 21). Questions on vital signs (i.e. fever) were excluded, since vital parameters are usually documented separately. The final expert-centered questionnaires comprised seven symptoms equally for AA and PNH (*fatigue, fever, bleeding tendency, dyspnea, pain, mood and concentration/memory disorders),* whereas the symptoms ‘*palpitations, tremors, muscle cramps and paresthesia/numbness’* were only included in the AA and ‘*dark urine, jaundice, dysphagia and erectile dysfunction’* in the PNH questionnaires.

According to the feedback of 8 AA and 4 PNH patients the final patient-centered questionnaires comprised seven matching symptoms (*fatigue, bleeding, dyspnea, mood and concentration/memory disorders, palpitations, and gastro-intestinal problems*). In addition, the symptoms ‘*hemoglobinuria*, *dysphagia*, *itching and pain*’ were incorporated for PNH, whereas ‘*muscle cramps, dizziness and trouble doing strenuous activities*’ for AA.

All questionnaires concluded with an open question to collect any further symptoms the patient experienced, resulting in a total of 12 (patient tailored AA questionnaire = 11) questionnaire items.

### Phase III (design of the PRO AA/PNH questionnaires)

The questions of the PRO-CTCAE (patient-reported outcome version of the Common Terminology Criteria for Adverse Events) [18] were considered most suitable for the majority of items identified by the previous consensus rounds. We also evaluated the use of questions from the EORTC-QLQ-C30 and FACIT-Fatigue, which have a main focus on HRQoL but covering less physical health. To still address HRQoL, which was a high priority for patients, we added regularly a sub-question on the interference with daily activities (see below).

**Fig. 3:**
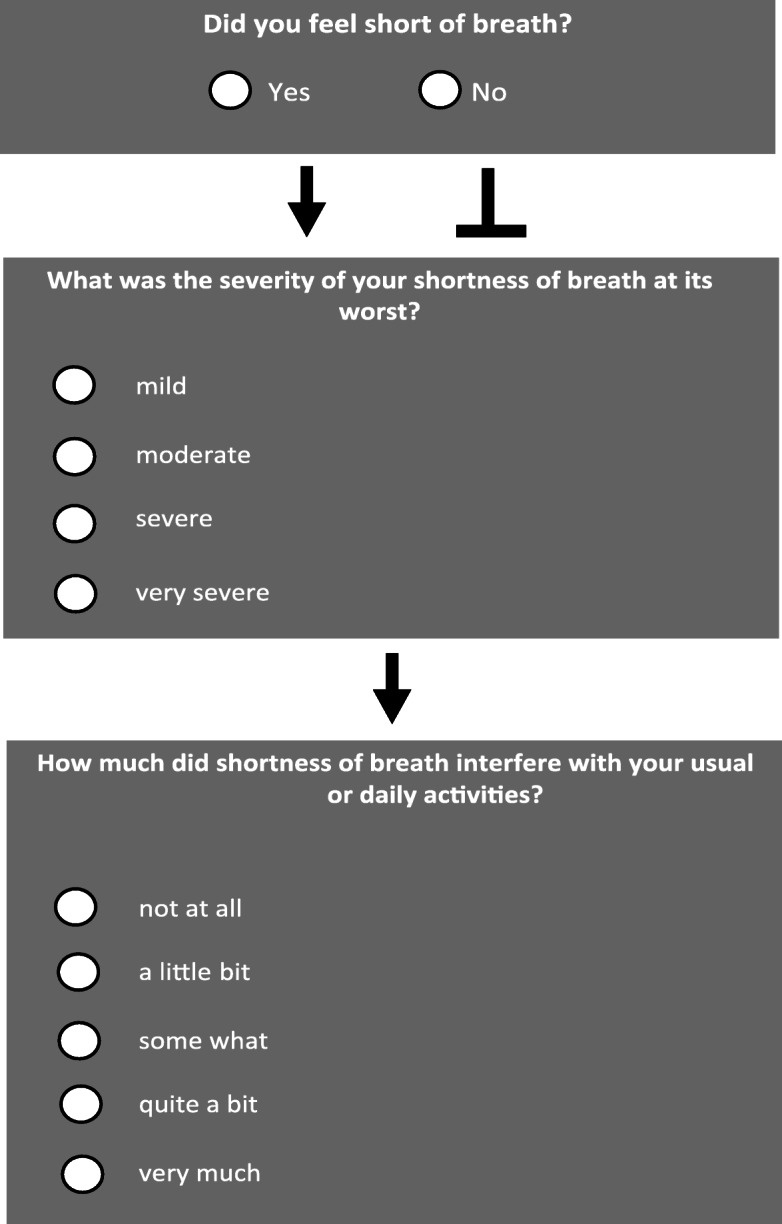
Example of a three-part symptom question

To minimize patient burden and assure greater completeness of data [17] we set-up an user-friendly structure for the questionnaire considering that patients might experience fatigue, psychosocial difficulties and time demands that make it inconvenient to answer long questionnaires. Since it is planned to incorporate the resulting PRO questionnaires into an electronic workflow for frequently monitoring symptoms (NCT04128943), a brief questionnaire was warranted. Based on these considerations, each symptom question was subdivided into three parts (Fig. 3):

1. The first part was constructed as a **‘yes/no’ – question** on patients’ symptom experience (e.g. *‘Did you feel short of breath?’*). In case a patient would answer with ‘no’, further sub-questions would not be visible and the anwering process could be shortened significantly.
2. Second the **severity of the symptom** (i.e. *mild, moderate, severe and very severe*) was addressed, following the format of the PRO-CTCAE questions.
3. Third, patients were queried on how much the symptom **interferes with their activities of daily living (HRQoL).**

For selected symptoms we decided for a modification of this design:

1. We changed the PRO-CTCAE question from ‘*general pain’* to ‘*pain*’ only, which facilitated the the further query of the location of pain.
2. For a better characterization of ‘*dysphagia*,’ we added a question on ‘*alterations in eating habits/swallowing*’, which is necessary for CTCAE version 5.0 grading but is not part of the PRO-CTCAE.
3. Due to the limited query of ‘*bleeding*’ in the PRO-CTCAE, we followed the modified WHO bleeding scale for locations and severity grading [13], which is highly reliable and strongly associated with platelet counts [33].
4. Since the PRO-CTCAE does not cover ‘*fever*’, we formulated a question relying on our institutional definition of fever based on Petersdorf et al. [34].
5. With regard to symptoms of ‘mood disorders’ (e.g. sad or unhappy feelings, anxiety, discourage) the PRO-CTCAE was regarded as incomplete. We therefore followed a validated two-question case-finding instrument with high sensitivity for depression by Whooley et al. [35].
6. None of the validated PRO questionnaires included questions on ‘*jaundice*’ and ‘*hemoglobinuria*’, therefore we phrased and graded both symptom questions based on personal clinical experience.
7. To cover gastro-intestinal problems, we combined the PRO-CTCAE questions ‘*nausea, vomiting, heartburn, gas, bloating, constipation and diarrhea*’.
8. “Troubles doing strenuous activities” were queried according to the EORTC-QLQ-C30 questionnaire.

Table 2 shows all items included in the final questionnaires.

**Table 2:** List of final questionnaire items and source

Questionnaire item	Source
Fatigue^a, b^	PRO-CTCAE Symptom Term: Fatigue
Fever^a^	Center specific diagnostic and treatment guidelines based on Petersdorf et al. [34]
Bleeding^a, b^	Modified WHO Bleeding Scale [13]
Dyspnea^a, b^	PRO-CTCAE Symptom Term: Shortness of breath
Pain^a^	According to PRO-CTCAE Symptom Term: General pain
Mood^a, b^	According to PRO-CTCAE Symptom Term: Sad; and based on an instrument by Whooley et al. [35]
Concentration/memory^a, b^	Combination of PRO-CTCAE Symptom Term: Concentration and PRO-CTCAE Symptom Term: Memory
Palpitations^a, b^	PRO-CTCAE Symptom Term: Heart palpitations
Gastro-intestinal problems^b^	PRO-CTCAE Symptom Terms: Nausea, Vomiting, Heartburn, Gas, Bloating, Constipation and Diarrhea
Open question on other symptom	N/A
Items only for AA questionnaire	
Palpitations^a^	PRO-CTCAE Symptom Term: Heart palpitations
Tremor^a^	According to CTCAE V5.0 Term: Tremor and adopted to PRO-CTCAE style
Muscle cramps^a, b^	According to CTCAE V5.0 Term: Muscle cramp and adopted to PRO-CTCAE style.
Paresthesia, numbness^a^	PRO-CTCAE Symptom Term: Numbness & tingling
Trouble doing strenuous activities^b^	EORTC-QLQ-C30
Dizziness^b^	PRO-CTCAE Symptom Term: Dizziness
Items only for PNH questionnaire	
Hemoglobinuria^a, b^	N/A
Jaundice^a^	N/A
Dysphagia^a, b^	According to CTCAE V5.0 Term: Dysphagia and adopted to the PRO-CTCAE Symptom Term: Difficulty swallowing
Erectile dysfunction^a^	PRO-CTCAE Symptom Term: Achieve and maintain erection
Itching^b^	PRO-CTCAE Symptom Term: Itching
Pain^b^	According to PRO-CTCAE Symptom Term: General pain

^a^experts choice; ^b^patients choice *WHO* World Health Organization, *PRO-CTCAE* patient-reported outcome version of the Common Terminology Criteria for Adverse Events, *EORTC-QLQ-C30* European Organization for Research and Treatment of Cancer Quality of Life of Cancer Patients, *N/A* not applicable

## Discussion

This study aimed to extract all patient-reported symptoms of AA/PNH in the literature and subsequently to identify the core symptoms including HRQoL by consensus rounds with patients and medical experts. The results formed the basis for the development of concise disease-specific PRO questionnaires for AA/PNH patients.

In the literature the most common reported symptoms addressed manifestations of pancytopenia in AA (e.g. fatigue, dyspnea, bleeding, fever) and hemolysis in PNH (e.g. abdominal pain, headache, dysphagia, erectile dysfunction). Since the standard method for documenting symptoms includes paper forms filled out by medical staff instead of patients directly, we assumed that many symptoms of AA/PNH might be underreported and the literature review is biased. In particular, data on HRQoL in AA/PNH was scarce and captured by questionnaires designed for cancer patients. For the assessment of physical constraints rarely a standardized query was performed, which emphasizes the need for AA/PNH specific tools for this matter. A first attempt to address this demand was recently made by a German group [5, 6], who developed a disease-specific HRQoL questionnaire together with patients. Our study aimed to expand this approach by designing a questionnaire not only for HRQoL but also for physical health in AA/PNH. Ultimately, the diversity of studies, including RCTs, observational studies and case reports/series made data extraction on PRO demanding since these sources were difficult to compare [36]. Accordingly, we observed differences in the frequency of recorded symptoms in case reports/series compared to observational studies and RCTs. Overall, identifying disease-specific symptoms in rare conditions in the literature is challenging and reinforced our strategy to include patients and medical experts for further identification of relevant symptoms.

Interestingly, the consensus rounds yielded only a few additional symptoms (e.g. tremor and ataxia, paresthesia, muscle spasms and cramps, impairment of family time). We assumed that our final symptom list was still not complete considering that the limited number of participating patients (*n* = 24) and medical experts (*n* = 7) cannot capture all aspects of this ultra-rare disease. This led us to add one open question in our final PRO questionnaire (“*Did you have any other symptoms/problems, which were not queried above*? ”) to capture potential missing symptoms with this questionnaire in the future.

By including patients and medical experts in the development of the PRO questionnaire we aimed to address the needs of both parties involved. Thereby, we observed significant differences in the symptom rating by experts compared to patients: patients judged HRQoL issues (e.g. fatigue, mood disorder, impaired activities of daily living) higher whereas experts favored physical constraints (e.g. fever, bleeding, pain, dyspnea, hemoglobinuria). This finding is consistent with previous studies, where patients consider symptoms associated with their daily health status as more important and clinicians focus on unfavorable clinical outcomes (e.g. emergency room admissions, mortality) in their assessments [37]. This different perspective by patients and medical experts emphasizes the need for tools addressing both sides and educating the parties on their different needs: On the one hand, patients can be instructed in understanding symptoms more in context of their disease with relevance for life-threat [38]. On the other hand, the physicians’ awareness of patients’ constraints in HRQoL can be enhanced. Disease-specific tools for self-reporting of symptoms are viable options for closing these gaps, as they support individualized care of patients, can improve patient-physician communication, clinical decision making and satisfaction with care, reportedly leading to a better outcome [10, 11, 39, 40]. However, our approach to combine patients’ and experts’ expectations was challenging for designing one PRO questionnaire that covers both demands while staying slim at the same time. In consequence, we drafted separate PRO questionnaires: *expert*-centered questionnaires focusing more on life-threatening events and complications (“red flags”) and *patient*-centered questionnaires covering less physical constraints and more HRQoL.

Up to now, PRO questionnaires have not been routinely used in the care of AA/PNH. We expect a benefit by these PRO tools since AA/PNH patients could easily report symptoms between and right before visits at specialized centers. Clinicians thereby could be informed more comprehensively on their patients’ disease burden, not only at the hospital but also in-between visits by electronically transmitted PRO questionnaires, which might complement care and treatment decisions in AA/PNH. Considering that family doctors and other medical disciplines are less familiar with these ultra-rare diseases and the overwhelming and unspecific information on the internet might confuse patients, the inclusion of PRO questionnaires within web-based workflows could help to guide AA/PNH patients by detecting relevant symptoms (including side effects), providing self-management instructions, educating on the disease and documenting medication intake with electronic tools. In the setting of PNH, this approach particularly might gain relevance as new complement inhibitors will be accessible for home administration and patients could be supported at home. For any investigator working in drug development or patient-centered outcome research this PRO instrument could also be useful for assessing symptoms and side effects in these rare conditions, in particular when comparing treatments in a head-to-head fashion (i.e. Eculizumab versus other complement inhibitors). Ultimately, there may also be a role for PROs in quality monitoring, which is increasingly demanded for regulatory reasons.

Based on these considerations and results of this study, we are now planning to include our final AA/PNH-specific PRO questionnaires into a web-based workflow for patient symptom-monitoring, which we intend to validate in a pursuing feasibility trial in AA/PNH patients (ClinicalTrials.gov: NCT04128943).

### Limitations

We are aware of several limitations. The term ‘PRO’ has just recently been defined; however, the screened literature often reports on symptoms in a non-systematic manner and it was not always clear if the extracted symptoms were reported directly by patients or reflected the indirect observation of the medical team. By this, the literature review might not only have been prone to bias due to underreporting of PRO, but also due to overrepresentation of some symptoms in the overall frequency. In several publications a partial overlap of the population was identified (i.e. population of the international and national PNH registries, population of the TRIUMPH and SHEPHERD trial). Therefore, the extracted study population might be overestimated and symptoms overrepresented. Since the differentiation of AA, PNH and AA-PNH syndrome is not always clear-cut, the separation of symptoms according to the sub-entities was difficult for further questionnaire design. In particular, patients with an AA-PNH overlap syndrome might not be covered by our AA or PNH PRO questionnaire. Based on the low incidence of the disease, we were not able to gather a large group of patients and medical experts for the consensus rounds, which might have been needed to identify more underreported symptoms.

## Conclusion

By literature review and consensus rounds with patients and medical experts it was feasible to develop disease-specific questionnaires for self-reporting of symptoms. These PRO questionnaires can now be validated within a web-based workflow in a subsequent feasibility study in AA/PNH.

## Supplementary information

**Additional file 1 Table S1.** keyword list for literature search. **Table S2.** search string for Embase. **Table S3.** inclusion and exclusion criteria - title/abstract screening. **Table S4.** inclusion and exclusion criteria - full-text screening. **Table S5.** Literature search: Studies reporting on clinical symptoms in AA (last update 05. May 2020). **Table S6.** Literature search: case reports and series reporting symptoms in AA patients (last update 05. May 2020). **Table S7.** Literature search: Studies reporting on clinical symptoms in PNH (last update 05. May 2020). **Table S8.** Literature search: case reports and series reporting symptoms in PNH patients (last update 05. May 2020). **Table S9.** Literature search: Studies reporting on clinical symptoms in AA-PNH. **Table S10.** Literature search: case reports and series reporting symptoms in AA-PNH patients. **Table S11.** Synthesis and frequency of PROs in observational studies and RCTs (last update 05. May 2020). **Table S12.** Synthesis and frequency of PROs in case reports and series (last update 05. May 2020). **Table S13.** symptom and QoL items included in the tentative questionnaire. **Table S14.** Patient characteristics of the patients evaluating the questionnaires. **Table S15.** mean ratings of questionnaire items by the patients: 1st Delphi round. **Table S16.** mean ratings of questionnaire items by the entire panel: 1st Delphi round. **Table S17.** comparison of AA questionnaire items rating: patients vs. experts. **Table S18.** comparison of PNH questionnaire items rating: patients vs. experts. **Table S19.** mean ratings of questionnaire items: 2nd Delphi round. **Table S20.** patients’ choice from the 3rd Delphi round. **Table S21.** experts’ modification of the questionnaire. **Table S22.** provisional PRO-AA/PNH questionnaire according to experts’ choice. **Table S23.** provisional patient centered PRO-AA/PNH questionnaire.

## Data Availability

All data generated or analyzed during this study are included in this published article and its supplementary file.
