# Supplementary material for: Development of a patient-reported outcome questionnaire for aplastic anemia and paroxysmal nocturnal hemoglobinuria (PRO-AA/PNH)
Source: Orphanet J Rare Dis. 2020 Sep 17;15:249. doi: 10.1186/s13023-020-01532-3 (PMC7495826; doi:10.1186/s13023-020-01532-3)
Supplement: Supplementary file 1 — Additional file 1 Table S1. keyword list for literature search. Table S2. search string for Embase. Table S3. inclusion and exclusion criteria - title/abstract screening. Table S4. inclusion and exclusion criteria - full-text screening. Table S5. Literature search: Studies reporting on clinical symptoms in AA (last update 05. May 2020). Table S6. Literature search: case reports and series reporting symptoms in AA patients (last update 05. May 2020). Table S7. Literature search: Studies reporting on clinical symptoms in PNH (last update 05. May 2020). Table S8. Literature search: case reports and series reporting symptoms in PNH patients (last update 05. May 2020). Table S9. Literature search: Studies reporting on clinical symptoms in AA-PNH. Table S10. Literature search: case reports and series reporting symptoms in AA-PNH patients. Table S11. Synthesis and frequency of PROs in observational studies and RCTs (last update 05. May 2020). Table S12. Synthesis and frequency of PROs in case reports and series (last update 05. May 2020). Table S13. symptom and QoL items included in the tentative questionnaire. Table S14. Patient characteristics of the patients evaluating the questionnaires. Table S15. mean ratings of questionnaire items by the patients: 1st Delphi round. Table S16. mean ratings of questionnaire items by the entire panel: 1st Delphi round. Table S17. comparison of AA questionnaire items rating: patients vs. experts. Table S18. comparison of PNH questionnaire items rating: patients vs. experts. Table S19. mean ratings of questionnaire items: 2nd Delphi round. Table S20. patients’ choice from the 3rd Delphi round. Table S21. experts’ modification of the questionnaire. Table S22. provisional PRO-AA/PNH questionnaire according to experts’ choice. Table S23. provisional patient centered PRO-AA/PNH questionnaire. [file 13023_2020_1532_MOESM1_ESM.docx]

**Supplementary material Patient-reported outcome questionnaire for aplastic anemia/paroxysmal nocturnal hemoglobinuria**

**List of keywords**

Table 1 keyword list for literature search

| Aplastic anemia | Side effects |
| --- | --- |
| Paroxysmal nocturnal hemoglobinuria | Adverse events |
| Bone marrow failure | Patient-reported symptoms |
|  | Patient-reported outcomes |
| Baseline characteristics | Self-reported outcome |
| Disease burden | Self-reported symptoms |
| Quality of life | Clinical outcomes |
| Health-related quality of life |  |
| Humans | Symptoms /Symptom |
| Clinical characteristics | Complications |
| Clinical course | Physical symptoms |
| Presenting features | Physical status |
| Clinical features | Signs / Sign |
| Clinical symptoms | Disorder |
| Clinical manifestations | Constitutional disorder |
| Clinical profile | Well-being |
| Physical concern | Presentation |
| Functional concern | Characteristics |
| Treatment-related concern | Somatic abnormalities |
| Clinical criteria |  |

**Search string**

Table 2 search string for Embase and Google Scholar

| **Search on Embase via OVID, 1980 to 2020 May 05; Search hits: 5222** |
| --- |
| (aplastic anemia/ OR aplastic anemia.ti,ab. OR aplastic anemias.ti,ab. OR paroxysmal nocturnal hemoglobinuria/ OR Paroxysmal Nocturnal Hemoglobinuria.ti,ab. OR Marchiafava-Micheli Syndrome.ti,ab. OR Bone marrow failure.ti,ab. AND (exp patient-reported outcome/ OR ((patient-reported.ti,ab. OR Self-reported.ti,ab. OR reported.ti,ab. OR clinical.ti,ab. OR clinically.ti,ab. OR physical.ti,ab. OR present$.ti,ab.) ADJ5 (symptom.ti,ab. OR symptoms.ti,ab. OR outcome.ti,ab. OR outcomes.ti,ab. OR features.ti,ab. OR feature.ti,ab. OR manifestations.ti,ab. OR disease burden.ti,ab. OR profile.ti,ab. OR criteria.ti,ab. OR status.ti,ab. OR sign.ti,ab. OR signs.ti,ab. OR presentation.ti,ab.)) OR suffer.ti,ab. OR suffered.ti,ab. OR suffering.ti,ab. OR quality of life.ti,ab. OR exp Quality of Life/ OR Side effect?.ti,ab. OR Adverse event?.ti,ab. OR adverse effect?.ti,ab. OR Complication?.ti,ab. OR Physical concern.ti,ab. OR Functional concern.ti,ab. OR Treatment-related concern.ti,ab. OR Well-being.ti,ab.) NOT (exp animal/ NOT exp human/) NOT (Comment.pt. OR Letter.pt. OR letter/ OR exp correspondence as topic/ OR Editorial.pt. OR editorial/ OR conference abstract.pt.)) |

# **Inclusion and exclusion criteria title/abstract screening**

Table 3 inclusion and exclusion criteria - title/abstract screening

| Item | inclusion-criteria (min. 1) | exclusion-criteria |
| --- | --- | --- |
| Title | PNH and/or paroxysmal nocturnal hemoglobinuria | absence of any inclusion-criteria |
|  | Marchiafava-Micheli syndrome | basic research |
|  | AA and/or aplastic anemia | histopathology |
|  | bone marrow failure | asymptomatic |
|  | anemia | animal studies |
|  | hemoglobinuria | laboratory analysis only |
|  | pancytopenia and/or bicytopenia | inherited bone marrow failure |
|  | allogeneic stem cell transplant | fanconi anemia |
|  | hematopoietic stem cell transplant and/or HSCT | dyskeratosis congenita |
|  | bone marrow transplantation | Shwachmann-Diamond syndrome |
|  | Eculizumab and/or complement inhibitor | Diamond-Blackfan anemia |
|  | anti-thymocyte globulin and/or ATG | telomeropathy |
|  | Eltrombopag |  |
|  | any description of a symptom |  |
|  | meeting any criteria from the keyword-list (see above) |  |
|  |  |  |
| Abstract | **inclusion criteria** | **exclusion criteria** |
|  | AA and/or aplastic anemia | animal research studies |
|  | PNH and/or paroxysmal nocturnal hemoglobinuria | basic research |
|  | bone marrow failure | not meeting any criteria from the keyword-list (see above) |
|  | pancytopenia | laboratory analysis only |
|  |  | no correlation between described symptom  and diagnosis of PNH and/or AA |
|  |  | cumulative study with < 10 patients included with the diagnosis of PNH and/or AA |

# **Inclusion criteria full-text screening**

Table 4 inclusion and exclusion criteria - full-text screening

| Inclusion criteria |
| --- |
| 1. Patient-reported symptom and/or recorded symptom, which could be described by patients |
| 1. Recorded number of patients reporting symptom or at least percentage |
| 1. Symptom recorded is relatable to the respective disease |
| 1. More than 10 patients in a subgroup (either PNH, AA or AA-PNH syndrome) analysis within a cohort study of diverse hematologic diseases (other than AA, PNH or AA-PNH) |
| Reasons for exclusion |
| 1. Language other than German or English |
| 1. Wrong population |
| 1. No (patient-reported) symptom described in the ‘result’ section or case presentation |
| 1. Less than 10 patients (either PNH, AA or AA-PNH syndrome) in a subgroup analysis within a cohort study of diverse hematologic diseases (other than AA, PNH or AA-PNH) |
| 1. Symptom not allocated to either PNH, AA and/or AA-PNH syndrome |

## **Publications reviewed (listed)**

Table 5 Literature search: Studies reporting on clinical symptoms in AA (last update 05. May 2020)

| Author | Year | Study design | Number of patients evaluated (n =) | Outcomes (No. of patients reporting symptom) |
| --- | --- | --- | --- | --- |
| Zhu | 2019 | prospective observational | 352 | minor bleeding (254) |
| Yamazaki | 2019 | prospective observational | 21 | major bleeding (1), minor bleeding (2), fever (3), hypertension (3), headache (3), urticaria (3), skin rash (2), back pain (4), abdominal pain (6), dyspepsia (2), nausea/vomiting (2), jaundice (1) |
| Shah | 2019 | retrospective observational | 91 | major bleeding (5), fever (52), hypertension (13), |
| Rogers | 2019 | retrospective observational | 314 | minor bleeding (75), |
| Lee | 2019 | prospective observational | 33 | minor bleeding (18), fatigue (16), urticaria (8), skin rash (4), arthralgia/myalgia (12), abdominal pain (2), dyspepsia (8), dizziness/vertigo (2), |
| Dutta | 2019 | retrospective and prospective observational | 76 | minor bleeding (56), pallor (69), |
| Hwang | 2018 | prospective observational | 20 | skin hyperpigmentation (16), abdominal discomfort (9) |
| Shah | 2018 | retrospective observational | 91 | fatigue (52), hypertension (13), gingival hyperplasia (14) |
| Suzuki | 2016 | retrospective observational | 47 | fever (7) |
| Ma | 2016 | retrospective observational | 77 | fever (19), hypertension (10), skin rash (4), arthralgia/myalgia (6) |
| Wei | 2015 | retrospective observational | 69 | fever (23), skin rash (23) |
| Kohgo | 2015 | prospective observational | 29 | skin rash (2), abdominal pain (4), nausea/vomiting (8), diarrhea (2), constipation (3), peripheral edema (3) |
| Kim | 2015 | prospective observational | 10 | fever (2), abdominal discomfort (2), nausea/vomiting (4), diarrhea (1) |
| Mahapatra | 2015 | retrospective observational | 1501 | minor bleeding (1044), fever (810), pallor (1456), jaundice (68) |
| Xue | 2015 | prospective observational | 20 | minor bleeding (1), fever (17), lymph node enlargement (1) |
| Hamad | 2014 | retrospective observational | 41 | fever (6), dyspnea (1), hypotension (1), skin rash (6) |
| Agarwal | 2014 | prospective observational | 30 | minor bleeding (3), fever (8), dyspnea (1), cough (1), headache (1), urticaria (4), arthralgia/myalgia (4), nausea/vomiting (2), jaundice (1), weight gain (1), blurred vision (1) |
| Wang | 2014 | retrospective observational | 56 | fever (5), dyspnea (1) |
| Shin | 2014 | retrospective observational | 61 | major bleeding (10), minor bleeding (41), dizziness/vertigo (3) |
| Jeong | 2014 | retrospective observational | 455 | major bleeding (6) |
| Gupta | 2014 | prospective observational | 175 | minor bleeding (145), fever (127), pallor (175) |
| Majnoon | 2014 | retrospective observational | 26 | minor bleeding (2), fever (12), skin rash (18), arthralgia/myalgia (1), weight loss (12), jaundice (3) |
| Wang | 2013 | prospective observational | 83 | nausea/vomiting (78), diarrhea (19), peripheral edema (11), hair loss (83), urinary retention (3) |
| Wang | 2013 | prospective observational | 22 | skin lesions (7) |
| Xiao | 2013 | prospective observational | 18 | fever (2), headache (1) |
| Gupta | 2013 | prospective observational | 66 | minor bleeding (10), fever (11), pallor (19) |
| Pawelec | 2013 | prospective observational | 123 | fever (25) |
| Chakrabarti | 2013 | prospective observational | 83 | minor bleeding (40), fatigue (83), fever (46), pallor (62) |
| Urban | 2012 | prospective observational | 18 | cough (1), skin rash (1) |
| Quarello | 2012 | retrospective observational | 78 | fever (51) |
| Wali | 2011 | retrospective observational | 90 | minor bleeding (80), fever (65), pallor (53) |
| Lee | 2010 | prospective observational | 116 | skin rash (13), abdominal pain (14), dyspepsia (9), nausea/vomiting (36), diarrhea (18), weight loss (7), hearing loss (1) |
| Kang | 2010 | prospective observational | 28 | minor bleeding (13), fever (8), dyspnea (3), skin rash (8), nausea/vomiting (18), diarrhea (12), weight gain (1), seizure (1) |
| Kwon | 2010 | retrospective observational | 96 | minor bleeding (30) |
| Park | 2010 | retrospective observational | 508 | major bleeding (86), minor bleeding (32) |
| Zheng | 2009 | retrospective observational | 57 | fever (36), skin rash (8), arthralgia/myalgia (5) |
| Malik | 2009 | prospective observational | 100 | minor bleeding (72), fatigue (25), fever (40), dyspnea (28), palpitation (30), pallor (32) |
| Buchholz | 2008 | retrospective observational | 20 | fever (2), lymphnode enlargement (2) |
| Pongtanakul | 2008 | retrospective observational | 42 | hypertension (5) |
| Hanif | 2007 | prospective observational | 44 | minor bleeding (43), mucosal ulcers (4) |
| Gupt | 2005 | prospective observational | 22 | fever (2), hypotension (2), skin rash (11), arthralgia/myalgia (9) |
| Goldenberg | 2004 | retrospective observational | 10 | fatigue (10), hypertension (1), urticaria (10) |
| Torres | 2003 | retrospective observational | 42 | major bleeding (3), fever (28) |
| Park | 2002 | prospective observational | 241 | arthralgia/myalgia (10) |
| Brennan | 2001 | retrospective observational | 79 | minor bleeding (34), fever (3), mucosal ulcers (12), gingival hyperplasia (13) |
| Fouladi | 2000 | retrospective observational | 46 | hypotension (8), diarrhea (3) |
| Mansour | 2000 | retrospective observational | 18 | blurred vision (7), loss of vision (2) |
| Sagmeister | 1999 | retrospective observational | 25 | major bleeding (8), blurred vision (1) |
| Trcuelu | 1998 | retrospective observational | 53 | fever (1), hypotension (3), urticaria (3), skin rash (13), arthralgia/myalgia (8) |
| Kelly | 1996 | prospective observational | 508 | minor bleeding (416), fatigue (399), fever (183), dyspnea (169), pallor (381) |
| Lopez | 1995 | prospective observational | 13 | fever (2), headache (3), urticaria (4), skin rash (5), diarrhea (1) |
| Sencer | 1993 | retrospective observational | 103 | minor bleeding (22) |
| Takahashi | 1993 | prospective observational | 37 | fatigue (4), fever (12), chest pain (1), headache (2), skin rash (2), arthralgia/myalgia (2), abdominal pain (1), nausea/vomiting (2), weight loss (2) |
| Doney | 1993 | prospective observational | 17 | fever (12), chills (1), headache (1), arthralgia/myalgia (1), nausea/vomiting (6), diarrhea (1) |
| Doney | 1992 | prospective observational | 68 | hypertension (20), chest pain (1), headache (2) |
| Frickhofer | 1991 | randomized controlled trial | 84 | hypertension (45), gingival hyperplasia (37), tremor (37), virilization (80) |
| Tichelli | 1988 | retrospecitve observational | 145 | major bleeding (12) |
| Hinterberger | 1987 | prospective observational | 23 | fever (13), arthralgia/myalgia (3), abdominal pain (1), diarrhea (1), hair loss (1), jaundice (2), dry mucous membranes (3) |
| Doney | 1987 | prospective observational | 46 | fever (23), dyspnea (1), hoarseness (4), hypertension (19), bradycardia (1), headache (2), skin rash (23), arthralgia/myalgia (24), virilization (3), seizure (1) |
| Hinterberger-Fischer | 1986 | prospective observational | 15 | fever (5), chills (5), hypertension (2), urticaria (3), skin rash (3), pruritus (3), back pain (3), arthralgia/myalgia (3), tremor (2) |
| Miller | 1983 | prospective observational | 19 | fever (11), chills (1), skin rash (10), arthralgia/myalgia (7), diarrhea (2), peripheral edema (2), cramps (1) |
| Mukiibi | 1981 | retrospective and prospective observational | 38 | minor bleeding (22), fatigue (33), fever (5), dyspnea (7), hypotension (1), palpitation (18), tachycardia (1), headache (13), pallor (2), mucosal ulcers (1), arthralgia/myalgia (9), peripheral edema (4), hemoglobinuria (1), jaundice (1), blurred vision (4), dizziness/vertigo (10) |

Table 6 Literature search: case reports and series reporting symptoms in AA patients (last update 05. May 2020)

| Author | Year | Number of patients evaluated (n =) | Outcomes (No. of patients reporting symptom) |
| --- | --- | --- | --- |
| Yamada | 2020 | 1 | minor bleeding (1) |
| Werum | 2020 | 1 | minor bleeding (1), fever (1), lymph node enlargement (1) |
| Suzuki | 2020 | 1 | minor bleeding (1), fatigue (1), pruritus (1) |
| Rosa | 2020 | 1 | minor bleeding (1), fever (1), diarrhea (1), nausea (1) |
| Yang | 2019 | 3 | major bleeding (3), pallor (3) |
| Su | 2019 | 6 | minor bleeding (2), fever (4), fatigue (1), abdominal pain (6); |
| Sahu | 2019 | 1 | fatigue (1), fever (1) |
| El-Cheikh | 2019 | 1 | minor bleeding (1), fever (1) |
| Ugarte-Torres | 2018 | 1 | minor bleeding (1), fever (1), pallor (1), arthralgia/myalgia (1), abdominal pain (1), diarrhea (1) |
| Irfan | 2018 | 1 | minor bleeding (1), fever (1), dyspnea (1), cough (1), pallor (1), skin rash (1), diarrhea (1) |
| Yang | 2018 | 1 | chest pain (1), tachycardia (1), pallor (1), abdominal distension (1), nausea/vomiting (1) |
| McGowan | 2018 | 12 | minor bleeding (4), fatigue (1), chest pain (1) |
| Hendren | 2017 | 1 | minor bleeding (1), fatigue (1), headache (1), arthralgia/myalgia (1) |
| Tao | 2017 | 1 | cough (1), hoarseness (1) |
| Kumar | 2017 | 1 | minor bleeding (1), fatigue (1), dyspnea (1), hypotension (1), tachycardia (1), pallor (1) |
| Shimizu | 2016 | 1 | fever (1), jaundice (1) |
| Suzuki | 2016 | 3 | minor bleeding (2), fever (1) |
| Nakano | 2016 | 1 | major bleeding (1), urinary retention (1) |
| Zekavat | 2016 | 1 | fever (1), headache (1) |
| Weng | 2016 | 2 | fever (2), diarrhea (1) |
| Vinod | 2016 | 1 | minor bleeding (1), fatigue (1), fever (1), skin rash (1), arthralgia/myalgia (1), abdominal pain (1) |
| John | 2016 | 1 | minor bleeding (1), fatigue (1), hypertension (1), pallor (1) |
| Scott | 2015 | 1 | fever (1) |
| Vo | 2015 | 2 | skin rash (1), skin lesions (1) |
| Lens | 2015 | 1 | fever (1), cough (1), sore throat (1) |
| Rathore | 2015 | 1 | seizure (1) |
| Kufelnicka-Babout | 2015 | 1 | major bleeding (1), minor bleeding (1), pallor (1) |
| Basu | 2014 | 1 | fatigue (1), fever (1), dyspnea (1), pallor (1), skin rash (1), abdominal pain (1), diarrhea (1) |
| Teichmann | 2014 | 1 | minor bleeding (1) |
| Miyashita | 2014 | 1 | dyspnea (1), skin hyperpigmentation (1), skin lesions (1), abdominal pain (1), nausea/vomiting (1) |
| Masferrer | 2014 | 1 | fever (1), arthralgia/myalgia (1) |
| Sharma | 2014 | 1 | minor bleeding (1), fatigue (1), pallor (1), seizure (1) |
| Ozdogu | 2014 | 1 | minor bleeding (1), fever (1), periorbital swelling (1) |
| Interiano | 2014 | 1 | major bleeding (1), fatigue (1), hypotension (1), tachycardia (1), syncope (1), pallor (1), abdominal pain (1), nausea/vomiting (1) |
| Kuriyama | 2014 | 1 | fever (1), lymph node enlargement (1) |
| Nakanishi | 2014 | 1 | fever (1), skin rash (1) |
| Hsu | 2014 | 1 | skin rash (1) |
| Grant | 2014 | 1 | skin rash (1) |
| Bozkaya | 2013 | 1 | minor bleeding (1), fever (1) |
| Munoz | 2013 | 1 | minor bleeding (1) |
| De Masson | 2013 | 4 | minor bleeding (2), fatigue (2), skin hyperpigmentation (1), pruritus (1), arthralgia/myalgia (4), weight loss (2) |
| Mitrovic | 2012 | 1 | fever (1), cough (1) |
| Alishiri | 2012 | 1 | minor bleeding (1), headache (1), arthralgia/myalgia (1), weight loss (1), dizziness/vertigo (1) |
| Ambey | 2012 | 1 | minor bleeding (1), fever (1), headache (1), pallor (1), nausea/vomiting (1), hemiparesis (1) |
| Bai | 2012 | 1 | abdominal discomfort (1) |
| Tanaka | 2012 | 1 | fever (1), lymph node enlargement (1) |
| Endo | 2012 | 1 | hypertension (1), headache (1), seizure (1) |
| Taylor | 2012 | 2 | minor bleeding (2), abdominal pain (1), weight loss (1), jaundice (1) |
| Marques | 2011 | 1 | fever (1), dyspnea (1), cough (1), odynophagia (1), headache (1) |
| Riaz Shah | 2011 | 1 | dyspnea (1), pallor (1), nausea/vomiting (1), diarrhea (1) |
| Ranganath | 2011 | 1 | minor bleeding (1), pale stool (1), hemoglobinuria (1), jaundice (1), loss of vision (1) |
| Rawlinson | 2011 | 1 | minor bleeding (1), fever (1), dyspnea (1), hypertension (1), hypotension (1), arthralgia/myalgia (1), abdominal pain (1) |
| Stibbe | 2011 | 1 | minor bleeding (1), fever (1), dyspnea (1), hypotension (1), headache (1), skin rash (1), nausea/vomiting (1), diarrhea (1) |
| Jose | 2011 | 1 | fever (1), dyspnea (1), hypertension (1), tachycardia (1), pallor (1), abdominal distension (1), jaundice (1) |
| Kuo | 2010 | 1 | fever (1), dyspnea (1), arthralgia/myalgia (1) |
| Al Nahdi | 2010 | 1 | skin rash (1) |
| Derber | 2010 | 1 | fever (1), headache (1), skin lesions (1) |
| Braakman | 2010 | 1 | hemiparesis (1) |
| Tschiedel | 2010 | 6 | nausea/vomiting (3), diarrhea (3), jaundice (6) |
| Agarwal | 2010 | 3 | loss of vision (3) |
| Al-Anazi | 2009 | 1 | minor bleeding (1), fever (1), pallor (1) |
| Koh | 2009 | 1 | minor bleeding (1) |
| Albuquerue | 2009 | 1 | major bleeding (1), fever (1), pallor (1) |
| Khetan | 2009 | 1 | blurred vision (1) |
| Nishikawa | 2009 | 1 | arthralgia/myalgia (1) |
| Agnihotri | 2009 | 1 | minor bleeding (1), pallor (1), gingival hyperplasia (1) |
| Albuquerque | 2009 | 1 | minor bleeding (1), fever (1), pallor (1) |
| Kim | 2008 | 1 | major bleeding (1) |
| Dolai | 2008 | 1 | fatigue (1), fever (1), dyspnea (1) |
| Al-Abdwani | 2008 | 1 | major bleeding (1), skin rash (1), abdominal pain (1), jaundice (1) |
| Celik | 2008 | 1 | minor bleeding (1), fatigue (1), pallor (1), skin rash (1) |
| Grey-Davies | 2008 | 3 | minor bleeding (1), fatigue (1), arthralgia/myalgia (1), abdominal pain (1), weight loss (1), jaundice (1) |
| Ghosh | 2008 | 3 | major bleeding (3), fatigue (1), fever (3), dyspnea (1), chest pain (2), pallor (1) |
| Chung | 2008 | 1 | fatigue (1), fever (1), sore throat (1), hypotension (1) |
| Ghosh | 2007 | 1 | loss of vision (1) |
| Mohla | 2006 | 1 | headache (1), pallor (1), blurred vision (1) |
| Fontaine | 2006 | 1 | fever (1), hypotension (1), lymph node enlargement (1) |
| Calistri | 2006 | 1 | minor bleeding (1), fever (1) |
| Itoh | 2006 | 1 | fever (1), cough (1), chest pain (1) |
| Ohga | 2006 | 1 | minor bleeding (1), pallor (1) |
| Jakucs and Pocsay | 2006 | 1 | major bleeding (1), fatigue (1), fever (1), dyspnea (1), odynophagia (1) |
| Sorensen | 2006 | 1 | fever (1), seizure (1) |
| Sharma | 2006 | 1 | abdominal pain (1) |
| Ghiasian | 2006 | 1 | fever (1), dyspnea (1), cough (1) |
| Nabavizadeh | 2006 | 1 | minor bleeding (1), fever (1), hypotension (1), arthralgia/myalgia (1) |
| Yeh | 2006 | 1 | jaundice (1), blurred vision (1), dry mucous membranes (1) |
| Petrikkos | 2006 | 2 | major bleeding (1), minor bleeding (1), fever (2), dyspnea (1), tachycardia (1), abdominal pain (2), jaundice (1) |
| Hansen | 2005 | 1 | minor bleeding (1) |
| Oyaizu | 2005 | 1 | minor bleeding (1), fatigue (1), pallor (1), gingival hyperplasia (1) |
| Nakano | 2004 | 1 | fever (1), pallor (1), diarrhea (1) |
| Yamamoto | 2004 | 1 | fever (1), odynophagia (1) |
| Park | 2003 | 1 | fever (1), dyspnea (1), cough (1) |
| Chuhjo | 2003 | 1 | fever (1), lymph node enlargement (1) |
| Choudhry | 2002 | 10 | minor bleeding (10), fatigue (3), fever (2), dyspnea (1), hypertension (1), pallor (7), diarrhea (1), blurred vision (1) |
| Jeng | 2002 | 2 | minor bleeding (1), fatigue (2), fever (1), hypertension (1), hypotension (1), bradycardia (1), headache (2), pallor (1), nausea/vomiting (2), blurred vision (1), photophobia (1), seizure (1) |
| Hayashi | 2001 | 1 | minor bleeding (1), abdominal pain (1) |
| Deka | 2001 | 2 | major bleeding (1), minor bleeding (1), fatigue (1), fever (1), pallor (1), loss of vision (1) |
| Mangione | 2000 | 1 | minor bleeding (1), fatigue (1), dyspnea (1), headache (1) |
| Holstein | 2000 | 1 | minor bleeding (1), fever (1), skin lesions (1) |
| Mao | 2000 | 1 | minor bleeding (1), pallor (1), skin rash (1), dizziness/vertigo (1) |
| Millar and Grammer | 2000 | 3 | minor bleeding (1), fatigue (1), fever (1), dyspnea (1), hypotension (1), chest pain (1), tachycardia (1), pruritus (1), nausea/vomiting (1), rigor (1) |
| Girmenia | 1999 | 1 | fever (1), hypertension (1), skin rash (1) |
| Oliviera | 1999 | 1 | dry mucous membranes (1) |
| Bahng | 1998 | 2 | fever (2), sore throat (2), dizziness/vertigo (1), rigor (1) |
| Richter | 1998 | 1 | minor bleeding (1), fatigue (1), palpitation (1) |
| Clark | 1997 | 1 | minor bleeding (1), fever (1), dyspnea (1), cough (1), sore throat (1), skin rash (1), nausea/vomiting (1) |
| Escobar-Morreale | 1997 | 1 | fever (1), sore throat (1) |
| Sato | 1997 | 1 | minor bleeding (1), skin rash (1), abdominal pain (1), diarrhea (1) |
| Kimo | 1997 | 4 | major bleeding (2), minor bleeding (2), fatigue (3), fever (1), skin thickening (2), weight loss (2) |
| Aquino | 1995 | 1 | minor bleeding (1), fever (1), dyspnea (1), tachycardia (1), headache (1), abdominal pain (1) |
| Zomas | 1995 | 1 | dyspnea (1) |
| Ogawa and Kanzaki | 1994 | 3 | dizziness/vertigo (3), hearing loss (3) |
| Ogawa and Kanzaki | 1994 | 3 | palpitation (1), headache (1), nausea/vomiting (2), dizziness/vertigo (3), hearing loss (3), tinnitus (3) |
| Weinblatt | 1991 | 2 | minor bleeding (2) |
| Blaser | 1989 | 1 | minor bleeding (1), skin thickening (1), weight loss (1) |
| Kojima | 1988 | 5 | hypertension (1), abdominal pain (1), diarrhea (1) |
| Kessler | 1988 | 1 | minor bleeding (1), fever (1), dyspnea (1), abdominal pain (1), nausea/vomiting (1), diarrhea (1), rectal pain (1), abnormal vaginal discharge (1) |
| Yeager | 1987 | 1 | abdominal pain (1), nausea/vomiting (1), diarrhea (1) |
| Baumgarten | 1987 | 1 | minor bleeding (1), pallor (1) |
| Mulholland and Delaney | 1983 | 4 | minor bleeding (2), fatigue (1), fever (4), hypotension (1), abdominal pain (4), nausea/vomiting (2), diarrhea (3) |
| Cairo and Baehner | 1982 | 4 | minor bleeding (4), fatigue (1), fever (1), headache (2), nausea/vomiting (1), blurred vision (1) |
| West | 1982 | 1 | major bleeding (1), fever (1), dyspnea (1), skin rash (1), abdominal pain (1), diarrhea (1) |
| Shiozwa | 1982 | 3 | headache (3), blurred vision (1), seizure (2), hemiparesis (3) |
| Bharucha | 1981 | 1 | minor bleeding (1), fatigue (1), fever (1), arthralgia/myalgia (1) |
| Tricot | 1980 | 1 | fever (1), cough (1), skin rash (1), abdominal pain (1), diarrhea (1), weight loss (1), dizziness/vertigo (1) |
| Suda | 1978 | 4 | major bleeding (1), minor bleeding (2), fever (1), dyspnea (1), palpitation (1), blurred vision (1) |

Table 7 Literature search: Studies reporting on clinical symptoms in PNH (last update 05. May 2020)

| Author | Year | Study design | Number of patients evaluated (n =) | Outcomes (No. of patients reporting symptom) |
| --- | --- | --- | --- | --- |
| Roth | 2020 | prospective observational | 29 | fatigue (3), fever (3), dyspnea (2), headache (5), back pain (3), abdominal pain (2), arthralgia/myalgia (3), diarrhea (4), urticaria (3), dizzines (4), dysuria (2) |
| Griesser | 2020 | retrospective observational | 150 | fatigue (22), abdominal pain (11) |
| Fu | 2020 | retrospective observational | 92 | minor bleeding (12), fatigue (72), dyspnea (39), hemoglobinuria (50); abdominal pain (21), headache (25), dysphagia (6), erectile dysfunction (12) |
| Sakurai | 2019 | retrospective observational | 1793 | minor bleeding (569), fatigue (1296), dyspnea (752), headache (720), back pain (327), dysphagia (258), abdominal pain (506), hemoglobinuria (700) |
| Lee | 2019 | randomized, controlled trial | 246 | fatigue (156), fever (19), dyspnea (80), cough (12), pharyngeal pain (14), chest pain (22), headache (85), arthralgia/myalgia (48), back pain (13), dysphagia (29), abdominal pain (46), dyspepsia (10), nausea/vomiting (21), diarrhea (15), hemoglobinuria (127), dizziness/vertigo (16), insomnia (8), erectile dysfunction (37) |
| Kulasekararaj | 2019 | randomized, controlled trial | 195 | fatigue (97), fever (14) abdominal pain (26), dyspnea (22), cough (15) dysphagia (4); chest pain (1), headache (43), arthralgia/myalgia (16), oropharyngeal pain (13), nausea and vomiting (27), diarrhea (16), constipation (12), hemoglobinuria (11), dizziness/vertigo (10), erectile dysfunction (12) |
| Patriquin | 2019 | prospective observational | 126 | fatigue (111), dyspnea (71), dysphagia (22), abdominal pain (55), hemoglobinuria (49), erectile dysfunction (13) |
| Devos | 2018 | prospective observational | 50 | minor bleeding (10), fatigue (41), dyspnea (18), dysphagia (1), abdominal pain (16), erectile dysfunction (3) |
| Choi | 2017 | prospective observational | 19 | dyspnea (11), chest pain (7), dysphagia (3), abdominal pain (14), hemoglobinuria (17), erectile dysfunction (6) |
| Wang | 2017 | retrospective observational | 24 | fatigue (18), dyspnea (15), abdominal pain (12), hemoglobinuria (18) |
| Mercuri | 2017 | retrospective observational | 6 | fatigue (6), headache (2), back pain (1), dysphagia (1), abdominal pain (4), hemoglobinuria (6), seizure (2) |
| Ninomiya | 2016 | prospective observational | 319 | fever (9), headache (138), arthralgia/myalgia (18), back pain (13), diarrhea (6), dizziness/vertigo (7) |
| Socie | 2016 | prospective observational | 1393 | fatigue (1012), dysphagia (210), abdominal pain (524), hemoglobinuria (767) |
| Kim | 2016 | retrospective observational | 162 | dyspnea (63), chest pain (21), abdominal pain (75), hemoglobinuria (99) |
| Chou | 2016 | prospective observational | 54 | fatigue (44), dyspnea (32), dysphagia (7), abdominal pain (17), hemoglobinuria (37), erectile dysfunction (17) |
| Sinan | 2016 | retrospective observational | 6 | fatigue (6), dyspnea (1), dysphagia (1), abdominal pain (1), hemoglobinuria (1) |
| Jang | 2016 | retrospective observational | 301 | dyspnea (112), chest pain (38), abdominal pain (142), hemoglobinuria (169) |
| Kelly | 2015 | prospective observational | 61 | major bleeding (10) |
| Sipol | 2015 | prospective observational | 2 | dysphagia (2), abdominal pain (2), hemoglobinuria (1) |
| Schrezenmeier | 2014 | prospective observational | 856 | fatigue (684), dyspnea (548), chest pain (282), headache (539), dysphagia (156), abdominal pain (368), hemoglobinuria (530), jaundice (256), dizziness/vertigo (228), erectile dysfunction (216) |
| Munoz-Linares | 2014 | retrospective observational | 56 | hypertension (18), dysphagia (16), abdominal pain (27), hemoglobinuria (34), erectile dysfunction (11) |
| Reiss | 2014 | prospective observational | 7 | minor bleeding (3), fatigue (2), dyspnea (1), headache (4), abdominal pain (3), reflux (2), nausea/vomiting (2), hemoglobinuria (4) |
| Hillmen | 2013 | prospective observational | 195 | minor bleeding (27), fatigue (25), fever (40), cough (39), sore throat (42), chest pain (6), headache (107), skin rash (20), pruritus (20), arthralgia/myalgia (111), back pain (48), abdominal pain (67), nausea/vomiting (113), diarrhea (68), peripheral edema (20), dizziness/vertigo (39), insomnia (23) |
| Dezern | 2013 | retrospective observational | 30 | fever (6) |
| Weitz | 2013 | prospective observational | 29 | fatigue (28), fever (19), headache (22), dysphagia (12), abdominal pain (17), hemoglobinuria (16), erectile dysfunction (9) |
| Kanakura | 2013 | prospective observational | 27 | minor bleeding (3), fever (4), headache (6), diarrhea (4) |
| Araten | 2012 | retrospective observational | 9 | minor bleeding (8), headache (1), abdominal distension (2), abdominal pain (4), nausea/vomiting (1), jaundice (2), loss of vision (1), hemiparesis (1) |
| Ge | 2012 | prospective observational | 50 | minor bleeding (7), fatigue (41), abdominal pain (4), hemoglobinuria (41) |
| Kanakura | 2011 | prospective observational | 29 | fever (5), headache (24), eczema (3), nausea/vomiting (15), diarrhea (7) |
| De Guibert | 2011 | prospective observational | 22 | fever (1) |
| Kelly | 2011 | prospective observational | 79 | dysphagia (9), abdominal pain (24), hemoglobinuria (50) |
| Brodsky | 2008 | prospective observational | 97 | fever (21), headache (52), arthralgia/myalgia (22), back pain (15), abdominal distension (1), abdominal pain (11), nausea/vomiting (30), diarrhea (12), dizziness/vertigo (14) |
| De Latour | 2008 | retrospective observational | 206 | abdominal pain (52) |
| Naithani | 2008 | retrospective observational | 18 | minor bleeding (6), fever (7), pallor (18), hemoglobinuria (7), jaundice (7), seizure (1) |
| Hillmen | 2006 | randomized, controlled trial | 115 | fatigue (6), fever (1), cough (9), headache (31), arthralgia/myalgia (8), back pain (12), abdominal pain (7), nausea/vomiting (7), diarrhea (9), dizziness/vertigo (7) |
| Hill | 2005 | prospective observational | 11 | cough (3), sore throat (4), arthralgia/myalgia (3), nausea/vomiting (3), hemoglobinuria (3) |
| Hillmen | 2004 | prospective observational | 11 | headache (4), arthralgia/myalgia (2), nausea/vomiting (3), dizziness/vertigo (3) |
| Boschetti | 2004 | retrospective observational | 23 | fever (1), arthralgia/myalgia (1), abdominal pain (2) |
| Hegenbart | 2003 | prospective observational | 7 | minor bleeding (1), fatigue (1), fever (2), syncope (1), arthralgia/myalgia (1), abdominal discomfort (1), abdominal pain (4), hemoglobinuria (1), seizure (1) |
| Mathieu | 1995 | prospective observational | 12 | major bleeding (1), abdominal pain (7) |
| Spath-Schwalbe | 1995 | prospective observational | 18 | minor bleeding (2), fatigue (10), abdominal pain (8), hemoglobinuria (11), jaundice (8) |
| Gongora-Biachi | 1993 | retrospective observational | 14 | minor bleeding (10), hemoglobinuria (4) |
| Koduri | 1992 | retrospective observational | 11 | major bleeding (1), fatigue (4), fever (4), abdominal pain (3), hemoglobinuria (7), jaundice (4) |
| Ware | 1991 | retrospective observational | 26 | fatigue (13), dyspnea (13), pallor (13), abdominal pain (10), hemoglobinuria (17), jaundice (2) |
| Saxena | 1991 | retrospective observational | 16 | minor bleeding (3), fatigue (7), pallor (16), abdominal pain (1), hemoglobinuria (7), jaundice (6) |
| Le | 1990 | retrospective observational | 476 | minor bleeding (206), abdominal pain (39), jaundice (1) |
| Parab | 1990 | retrospective observational | 17 | minor bleeding (13), fever (8), jaundice (8) |
| Forman | 1984 | retrospective observational | 26 | minor bleeding (2), fatigue (14), fever (1), dyspnea (3), chest pain (1), headache (3), pallor (4), arthralgia/myalgia (1), abdominal pain (5), weight loss (2), hemoglobinuria (11), jaundice (6) |

Table 8 Literature search: case reports and series reporting symptoms in PNH patients (last update 05. May 2020)

| Author | Year | Number of patients evaluated (n =) | Outcomes (No. of patients reporting symptom) |
| --- | --- | --- | --- |
| Sanchez-Petitto | 2020 | 1 | abdominal pain (1), hemoglobinuria (1), syncope (1) |
| Zhang | 2019 | 1 | fatigue (1), hemoglobinuria (1), dizziness/vertigo |
| Rosa | 2019 | 1 | minor bleeding (1), fatigue (1), palpitations (1) |
| Rodriguez-Ferreras and Velasco-Roces | 2019 | 1 | minor bleeding (1), fatigue (1), chest pain (1), abdominal pain (1) |
| Rahman and Chowdhury | 2019 | 1 | fatigue (1), pallor (1), fever (1), cough (1), hemoglobinuria (1), mucosal ulcers (1) |
| Nguyen | 2019 | 1 | minor bleeding (1), fatigue (1), arthralgia/myalgia (1) |
| Hochsmann | 2019 | 4 | fever (2), headache (2), arthralgia/myalgia (5), abdominal pain (2), urticaria (3) |
| Elias | 2018 | 2 | minor bleeding (1), abdominal pain (2), hemoglobinuria (1) |
| Bastos | 2018 | 1 | fatigue (1), abdominal discomfort (1), nausea/vomiting (1), hemoglobinuria (1) |
| Mancuso | 2018 | 1 | fatigue (1), fever (1), abdominal pain (1), nausea/vomiting (1), loss of appetite (1), hemoglobinuria (1) |
| Rao | 2018 | 1 | hypertension (1), pallor (1), nausea/vomiting (1), peripheral edema (1), oliguria (1), hemoglobinuria (1) |
| Lauritsch-Hernandez | 2018 | 1 | major bleeding (1), fatigue (1), fever (1), hypertension (1) |
| Dragoni | 2018 | 5 | major bleeding (1) |
| Zekria | 2017 | 1 | fever (1), tachycardia (1), abdominal pain (1), diarrhea (1), loss of appetite (1) |
| Kabir | 2017 | 1 | fatigue (1), fever (1), dyspnea (1), cough (1), hypotension (1), chest pain (1), headache (1), arthralgia/myalgia (1), nausea/vomiting (1), peripheral edema (1) |
| Thanos | 2017 | 1 | blurred vision (1) |
| Puri | 2017 | 3 | fatigue (1), fever (2), dyspnea (1), pallor (3), peripheral edema (3), oliguria (1), jaundice (1) |
| Quinquenel | 2017 | 1 | fatigue (1), fever (1), chest pain (1) |
| Ram | 2017 | 14 | minor bleeding (2), reflux (2) |
| De-la-Iglesia | 2016 | 1 | major bleeding (1), fatigue (1), fever (1), syncope (1), headache (1), erectile dysfunction (1) |
| Roth | 2015 | 1 | fatigue (1), hemoglobinuria (1), jaundice (1) |
| Ding | 2015 | 1 | hypertension (1), headache (1), seizure (1) |
| Sahin | 2015 | 3 | fatigue (2), abdominal pain (1), nausea/vomiting (1), jaundice (1) |
| Figueroa-Jimenez | 2015 | 1 | fatigue (1), hypotension (1), abdominal discomfort (1), reflux (1), nausea/vomiting (1), hemoglobinuria (1) |
| Gessoni | 2015 | 1 | dyspnea (1), abdominal pain (1) |
| Ahluwalia | 2014 | 2 | minor bleeding (2), fever (1), abdominal pain (1) |
| Pande | 2014 | 1 | major bleeding (1), fatigue (1), fever (1), hemoglobinuria (1), jaundice (1) |
| Mason | 2014 | 1 | minor bleeding (1), cough (1), hypertension (1), headache (1), abdominal pain (1), nausea/vomiting (1), hemoglobinuria (1), jaundice (1), blurred vision (1), dizziness/vertigo (1) |
| Tezcaner | 2014 | 1 | minor bleeding (1), fatigue (1), abdominal pain (1), nausea/vomiting (1), weight loss (1) |
| Parveen | 2014 | 1 | fatigue (1), fever (1), hypertension (1), abdominal pain (1), nausea/vomiting (1), weight loss (1), hemoglobinuria (1), jaundice (1) |
| Memon | 2014 | 1 | headache (1), dysphagia (1), nausea/vomiting (1), hemoglobinuria (1), blurred vision (1), seizure (1) |
| Crawford | 2014 | 1 | major bleeding (1), fever (1), hypertension (1), skin lesions (1) |
| Araten | 2014 | 4 | minor bleeding (2), fatigue (1), fever (1), headache (1), abdominal pain (3) |
| Mandala | 2013 | 1 | fatigue (1), pallor (1), back pain (1), abdominal distension (1), abdominal pain (1), jaundice (1) |
| Townsley and Young | 2013 | 1 | fever (1), pallor (1), abdominal pain (1), nausea/vomiting (1), hemoglobinuria (1) |
| Hayakawa | 2013 | 1 | fatigue (1), fever (1) |
| De Boysson | 2013 | 1 | arthralgia/myalgia (1), weight loss (1) |
| Wijewickrama | 2013 | 1 | fever (1), oliguria (1) |
| Yang | 2013 | 2 | tachycardia (1), pallor (2), loss of vision (2) |
| Brodsky | 2012 | 1 | fatigue (1), headache (1), abdominal distension (1), abdominal pain (1), nausea/vomiting (1) |
| Kim | 2012 | 1 | fever (1), cough (1) |
| Nakamura | 2011 | 1 | fatigue (1), headache (1), arthralgia/myalgia (1), nausea/vomiting (1), hemoglobinuria (1) |
| Al-Sayes | 2010 | 1 | fatigue (1), dyspnea (1), chest pain (1), pallor (1), abdominal pain (1), hemoglobinuria (1), jaundice (1), dizziness/vertigo (1) |
| Naseem | 2009 | 1 | hemoglobinuria (1), jaundice (1) |
| Uzun | 2008 | 1 | fatigue (1), fever (1), abdominal pain (1), weight loss (1) |
| Misra | 2008 | 1 | major bleeding (1), fever (1), headache (1), abdominal distension (1), nausea/vomiting (1), jaundice (1), blurred vision (1), hemiparesis (1) |
| Samadder | 2007 | 1 | nausea/vomiting (1), hemoglobinuria (1), dizziness/vertigo (1) |
| Allen | 2007 | 1 | fatigue (1), dyspnea (1), hypertension (1) |
| Fieni | 2006 | 1 | fever (1), hypotension (1), back pain (1) |
| Chen | 2006 | 1 | fatigue (1), hypertension (1) |
| Kumpers | 2006 | 1 | major bleeding (1), dyspnea (1), hypertension (1), hypotension (1), diarrhea (1) |
| Sharma | 2005 | 1 | fever (1), headache (1), pallor (1), abdominal distension (1), jaundice (1), blurred vision (1), hemiparesis (1) |
| Millonig | 2004 | 1 | major bleeding (1), abdominal distension (1), abdominal pain (1), jaundice (1) |
| Bahr | 2003 | 1 | major bleeding (1), headache (1), abdominal discomfort (1), seizure (1) |
| Lee | 2003 | 1 | minor bleeding (1), skin rash (1), skin lesions (1), abdominal pain (1), peripheral edema (1), dry mucous membrane (1) |
| Bjorge | 2003 | 1 | minor bleeding (1), fatigue (1), fever (1), hypertension (1), abdominal distension (1), abdominal pain (1), peripheral edema (1) |
| Liebman and Feinstein | 2003 | 2 | headache (1), abdominal distension (1), abdominal pain (1), hemiparesis (1) |
| Adams | 2002 | 1 | minor bleeding (1), fatigue (1), hypotension (1), abdominal pain (1), nausea/vomiting (1), hemoglobinuria (1), jaundice (1) |
| Kanegusuku | 2001 | 1 | minor bleeding (1) |
| Gayer | 2001 | 10 | minor bleeding (2), fatigue (1), fever (3), dyspnea (1), dysphagia (1), abdominal distension (1), abdominal pain (7), nausea emesis (2), jaundice (2), blurred vision (1) |
| Chow | 2001 | 1 | pallor (1), arthralgia/myalgia (1), hemoglobinuria (1), jaundice (1) |
| Doukas | 1998 | 1 | minor bleeding (1), pallor (1), jaundice (1) |
| Hauser | 1996 | 1 | blurred vision (1) |
| Graham | 1996 | 1 | fever (1), mucosal ulcers (1), abdominal pain (1), nausea/vomiting (1), jaundice (1) |
| Huong | 1995 | 1 | abdominal pain (1), hemoglobinuria (1), jaundice (1) |
| Al-Samman | 1994 | 1 | fatigue (1), fever (1), dyspnea (1), cough (1), headache (1), hemoglobinuria (1), jaundice (1), hemiparesis (1), aphasia (1) |
| Imai | 1989 | 1 | minor bleeding (1), fever (1), abdominal pain (1), peripheral edema (1), hemoglobinuria (1), hemiparesis (1) |
| Klein and Hartmann | 1989 | 1 | chest pain (1), arthralgia/myalgia (1), abdominal pain (1), peripheral edema (1), jaundice (1) |
| Takagi | 1989 | 1 | fever (1), abdominal pain (1), peripheral edema (1), oliguria (1), hemoglobinuria (1), hemiparesis (1) |
| Schmid | 1989 | 1 | major bleeding (1), headache (1), abdominal distension (1), abdominal discomfort (1), seizure (1), aphasia (1) |
| Valla | 1987 | 5 | major bleeding (1), minor bleeding (2), fever (5), abdominal distension (3), abdominal pain (5) |
| Vellenga | 1982 | 2 | abdominal pain (2) |
| Stirling | 1980 | 1 | headache (1), hemiparesis (1), neck stiffness (1), paresthesia (1) |
| Hartmann | 1980 | 1 | fever (1), abdominal distension (1), abdominal pain (1), nausea/vomiting (1), hemoglobinuria (1) |
| Stirling | 1980 | 1 | headache (1), hemoglobinuria (1), hemiparesis (1), neck stiffness (1), paresthesia (1) |

Table 9 Literature search: Studies reporting on clinical symptoms in AA-PNH

| Author | Year | Study design | Number of patients evaluated (n =) | Outcomes (No. of patients reporting symptom) |
| --- | --- | --- | --- | --- |
| Choi | 2017 | prospective observational | 27 | dyspnea (13), chest pain (5), dysphagia (9), abdominal pain (18), hemoglobinuria (21) |
| Mercuri | 2017 | retrospective observational | 5 | minor bleeding (1), fatigue (3), chest pain (1), headache (2), back pain (3), abdominal pain (2), hemoglobinuria (2) |
| Socie | 2016 | prospective observational | 375 | fatigue (290), dysphagia (43), abdominal pain (332), hemoglobinuria (100) |
| Kim | 2016 | retrospective observational | 120 | dyspnea (46), chest pain (17), abdominal pain (62), hemoglobinuria (66) |
| Sipol | 2015 | prospective observational | 5 | fatigue (1), dysphagia (1), abdominal pain (1), hemoglobinuria (1) |
| Ge* | 2015 | retrospective observational | 55 | minor bleeding (46), fatigue (49), hemoglobinuria (2) |
| De Azambuja* | 2015 | retrospective observational | 103 | minor bleeding (33), fatigue (98), abdominal pain (33), hemoglobinuria (19), jaundice (26) |
| Curran* | 2012 | retrospective observational | 12 | minor bleeding (5), fatigue (9), dyspnea (9), pallor (9), hemoglobinuria (1) |
| Ge | 2012 | prospective observational | 230 | minor bleeding (164), fatigue (209), abdominal pain (1), hemoglobinuria (32) |
| De Latour | 2008 | retrospective observational | 224 | abdominal pain (27) |
| Moyo* | 2004 | retrospective observational | 49 | fatigue (41), headache (12), abdominal pain (17), hemoglobinuria (21), erectile dysfunction (7) |
| Spath-Schwalbe | 1995 | prospective observational | 22 | minor bleeding (19), fatigue (17), hemoglobinuria (5), jaundice (2) |

*majority of patients evaluated with AA-PNH syndrome

Table 10 Literature search: case reports and series reporting symptoms in AA-PNH patients

| Author | Year | Number of patients evaluated (n =) | Outcomes (No. of patients reporting symptom) |
| --- | --- | --- | --- |
| Kogiso | 2016 | 1 | abdominal distension (1), weight loss (1) |
| Pan | 2014 | 1 | fever (1), skin lesions (1) |
| Martin-Diaz | 2012 | 1 | minor bleeding (1), hemoglobinuria (1) |
| Chen | 2007 | 1 | minor bleeding (1), pallor (1), oliguria (1), hemoglobinuria (1), jaundice (1) |
| Audebert | 2005 | 2 | minor bleeding (1), fatigue (2), fever (1), dyspnea (1), jaundice (1), seizure (1), hemiparesis (1) |
| Munte and Muller-Vahl | 1995 | 1 | minor bleeding (1), hypertension (1), headache (1), arthralgia myalgia (1), abdominal pain (1), hemoglobinuria (1), seizure (1), hemiparesis (1) |

# **Frequency distribution of patient-reported symptoms**

Table 11 Synthesis and frequency of PROs in observational studies and RCTs (last update 05. May 2020)

| Clinical features | Patients reporting symptom in %  (in relation to total no. of patients of the respective disease) | | |
| --- | --- | --- | --- |
|  | AA (n = 6851) | PNH (n = 7647) | AA-PNH (n= 1227) |
| Minor bleeding (WHO 1 - 2) | 35.94 | 11.53 | ─ |
| Pallor | 32.83 | 0.67 | 0.73 |
| Fever | 24.48 | 2.16 | ─ |
| Fatigue | 9.08 | 49.85 | 58.44 |
| Dyspnea | 3.08 | 23.41 | 5.54 |
| Nausea/vomiting | 2.28 | 2.90 | ─ |
| Skin rash | 2.28 | 0.26 | ─ |
| Major bleeding (WHO 3 - 4) | 1.91 | 0.16 | 21.84 |
| Hypertension | 1.91 | 0.24 | ─ |
| Arthralgia/myalgia | 1.52 | 3.06 | ─ |
| Hair loss | 1.23 | ─ | ─ |
| Virilization | 1.21 | ─ | ─ |
| Gingival hyperplasia | 1.15 | ─ | ─ |
| Jaundice | 1.11 | 3.92 | 2.28 |
| Diarrhea | 0.88 | 1.84 | ─ |
| Palpitation | 0.70 | ─ | ─ |
| Tremor | 0.57 | ─ | ─ |
| Urticaria | 0.51 | ─ | ─ |
| Headache | 0.41 | 23.68 | 1.14 |
| Abdominal pain | 0.41 | 28.66 | 40.18 |
| Mucosal ulcers | 0.38 | ─ | ─ |
| Weight loss | 0.31 | 0.03 | ─ |
| Peripheral edema | 0.29 | 0.26 | ─ |
| Dyspepsia/reflux | 0.28 | 0.16 | ─ |
| Skin hyperpigmentation | 0.23 | ─ | ─ |
| Hypotension | 0.22 | ─ | ─ |
| Dizziness/vertigo/vertigo | 0.22 | 4.29 | ─ |
| Blurred vision | 0.19 | ─ | ─ |
| Abdominal discomfort | 0.16 | 0.04 | ─ |
| Chills | 0.10 | ─ | ─ |
| Skin lesions | 0.10 | ─ | ─ |
| Back pain | 0.10 | 5.65 | 0.24 |
| Hoarseness | 0.06 | ─ | ─ |
| Pruritus | 0.04 | 0.26 | ─ |
| Constipation | 0.04 | ─ | ─ |
| Urinary retention | 0.04 | ─ | ─ |
| Lymphnode enlargement | 0.04 | ─ | ─ |
| Dry mucous membranes | 0.04 | ─ | ─ |
| Chest pain | 0.03 | 4.94 | 1.87 |
| Cough | 0.03 | 1.02 | ─ |
| Seizure | 0.03 | 0.05 | ─ |
| Loss of vision | 0.03 | 0.01 | ─ |
| Weight gain | 0.03 | ─ | ─ |
| Hemoglobinuria | 0.01 | 37.19 | 22.00 |
| Cramps | 0.01 | ─ | ─ |
| Bradycardia | 0.01 | ─ | ─ |
| Tachycardia | 0.01 | ─ | ─ |
| Hearing loss | 0.01 | ─ | ─ |
| Dysphagia | ─ | 9.93 | 4.32 |
| Erectile dysfunction | ─ | 4.56 | 0.57 |
| Sore throat | ─ | 0.95 | ─ |
| Insomnia | ─ | 0.41 | ─ |
| Abdominal distension | ─ | 0.01 | ─ |
| Eczema | ─ | 0.04 | ─ |
| Syncope | ─ | 0.01 | ─ |
| Hemiparesis | ─ | 0.01 | ─ |
| Dysuria | ─ | 0.03 |  |

Table 12 Synthesis and frequency of PROs in case reports and series (last update 05. May 2020)

| Clinical features | Patients reporting symptom in %  (in relation to total no. of patients of the respective disease) | | |
| --- | --- | --- | --- |
|  | AA (n = 201) | PNH (n = 120) | AA-PNH (n = 7) |
| Minor bleeding (WHO 1 - 2) | 41.29 | 18.85 | 57.1 |
| Fever | 38.31 | 31.15 | 28.6 |
| Pallor | 17.91 | 10.66 | 14.3 |
| Fatigue | 17.41 | 26.23 | 28.6 |
| Dyspnea | 12.44 | 6.56 | 14.3 |
| Abdominal pain | 14.93 | 40.16 | 14.3 |
| Nausea/vomiting | 10.45 | 16.39 | ─ |
| Diarrhea | 11.44 | 1.64 | ─ |
| Headache | 9.45 | 14.75 | 14.3 |
| Skin rash | 8.96 | 0.82 | ─ |
| Major bleeding (WHO 3 - 4) | 9.45 | 9.02 | ─ |
| Arthralgia/myalgia | 7.46 | 9.02 | 14.3 |
| Jaundice | 6.97 | 17.21 | 28.6 |
| Cough | 4.98 | 4.1 | ─ |
| Hypotension | 4.98 | 4.1 | ─ |
| Dizziness/vertigo/vertigo | 4.98 | 3.28 | ─ |
| Weight loss | 4.98 | 3.28 | 14.3 |
| Hypertension | 3.98 | 8.20 | 14.3 |
| Blurred vision | 3.98 | 5.74 | ─ |
| Tachycardia | 3.48 | 1.64 | ─ |
| Seizure | 3.48 | 3.28 | 28.6 |
| Sore throat | 2.99 | ─ | ─ |
| Chest pain | 2.99 | 4.1 | ─ |
| Loss of vision | 2.99 | 1.64 | ─ |
| Hearing loss | 2.99 | ─ | ─ |
| Hemiparesis | 2.49 | 6.56 | 28.6 |
| Skin lesions | 1.99 | 1.64 | 14.3 |
| Lymph node enlargement | 2.49 | ─ | ─ |
| Odynophagia | 1.49 | ─ | ─ |
| Palpitation | 1.49 | 0.82 | ─ |
| Skin thickening | 1.49 | ─ | ─ |
| Tinnitus | 1.49 | ─ | ─ |
| Skin hyperpigmentation | 1.00 | ─ | ─ |
| Pruritus | 1.49 | ─ | ─ |
| Abdominal distension | 1.00 | 10.66 | 14.3 |
| Gingival hyperplasia | 1.00 | ─ | ─ |
| Dry mucous membranes | 1.00 | 0.82 | ─ |
| Rigor | 1.00 | ─ | ─ |
| Hoarseness | 0.50 | ─ | ─ |
| Bradycardia | 0.50. | ─ | ─ |
| Syncope | 0.50 | 1.64 | ─ |
| Abdominal discomfort | 0.50 | 3.28 | ─ |
| Urinary retention | 0.50 | ─ | ─ |
| Pale stool | 0.50 | ─ | ─ |
| Hemoglobinuria | 0.50 | 22.13 | 42.9 |
| Photophobia | 0.50 | ─ | ─ |
| Periorbital swelling/pain | 0.50 | ─ | ─ |
| Rectal pain/tenesmus | 0.50 | ─ | ─ |
| Peripheral edema | ─ | 8.20 | ─ |
| Oliguria | ─ | 3.28 | 14.3 |
| Dyspepsia/reflux | ─ | 2.46 | ─ |
| Paresthesia | ─ | 1.64 | ─ |
| Aphasia | ─ | 1.64 | ─ |
| Neck stiffness | ─ | 1.64 | ─ |
| Dysphagia | ─ | 1.64 | ─ |
| Back pain | ─ | 1.64 | ─ |
| Loss of appetite | ─ | 1.64 | ─ |
| Erectile dysfunction | ─ | 0.82 | ─ |
| Mucosal ulcers | ─ | 1.64 | ─ |

# **List of symptoms and QoL issues included in the tentative questionnaire**

Table 13 symptom and QoL items included in the tentative questionnaire

| Aplastic anemia | PNH |
| --- | --- |
| Fatigue | Fatigue |
| Bleeding | Bleeding |
| Pain | Pain |
| Dyspnea | Dyspnea |
| Cough | Cough |
| Gastrointestinal disorder | Gastrointestinal disorder |
| Skin alteration | Dysphagia |
| Peripheral edema | Skin alteration |
| Icterus/jaundice | Peripheral edema |
| Fever | Dark urine/hemoglobinuria |
| Hypo-/hypertension | Icterus/jaundice |
| Brady-/tachycardia | Fever |
| Palpitation | Hypo-/hypertension |
| Weight gain/loss | Brady-/tachycardia |
| Changes in sensory perception | Palpitation |
| Dizziness/vertigo | Weight gain/loss |
| Pruritus | Changes in sensory perception |
| Insomnia | Dizziness/vertigo |
| Concentration disorder | Pruritus |
| Depression* | Erectile dysfunction |
| Worry* | Insomnia |
| Tense feeling* | Concentration disorder |
| Impaired at work or in ADL* | Depression |
| Impaired leisure time* | Worry* |
| Impaired performance in strenuous activities* | Tense feeling* |
| Impaired performance in walking long distances* | Impaired at work or in ADL* |
| Spending time in bed or chair during the day* | Impaired leisure time* |
|  | Impaired performance in strenuous activities* |
|  | Impaired performance in walking long distances* |
|  | Spending time in bed or chair during the day* |
| *questions on quality of life according to EORTC Quality of Life Group (1995). EORTC QLQ-C30 v3.0. ADL: activities of daily living | |

# **Results of the 1^st^ and 2^nd^ Delphi rounds**

Table 14 Patient characteristics of the patients evaluating the questionnaires

|  | 1^st^ Delphi round | |  |
| --- | --- | --- | --- |
|  | AA (n = 13) | PNH (n = 8) | AA-PNH (n = 3) |
| Sex |  |  |  |
| Female | 6 | 4 | 2 |
| Male | 7 | 4 | 1 |
| Age (in years) |  |  |  |
| Mean; range | 54.4; 25 – 82 | 39.3; 21 – 64 | 36.0; 26 – 51 |

Table 15 mean ratings of questionnaire items by the patients: 1st Delphi round

| Questionnaire items from the 1^st^ Delphi round | Patient rating (mean) |
| --- | --- |
|  | AA; PNH |
| 1. In general, do you feel tired? | 3.6; 3.9 |
| 1. Do you have an increased bleeding tendency? | 3.0; 2.4 |
| 1. Have you been in pain? | 2.9; 3.4 |
| 1. Did you experience shortness of breath? | 3.3; 3.2 |
| 1. Did you have a cough? | 2.4; 2.3 |
| 1. Did you have digestive/gastrointestinal problems? | 2.6; 2.9 |
| 1. Did you have difficulties in swallowing things? | N/A; 2.5 |
| 1. Have you noticed changes in hair, skin and/or mucous membranes? | 2.8; 2.3 |
| 1. Did you have swelling/oedema of your limbs? | 2.4; 1.9 |
| 1. Have you noticed a dark discolouration of the urine? | N/A; 3.5 |
| 1. Have you noticed a yellowish discoloration of your ‘white of the eye’? | 2.2; 2.8 |
| 1. Did you have fever (from 38.0°C)? | 2.6; 2.7 |
| 1. Did you record a high blood pressure? (upper value >140 mmHg, lower value >80 mmHg) | 2.8; 2.5 |
| 1. Did you record a too low or to high pulse? (< 60 beats/minute or >90 beats/minute) | 2.9; 2.5 |
| 1. Did you experience palpitations? (unpleasant sensation of irregular and/or forceful beating of the heart) | 2.4; 2.7 |
| 1. Did you lose or gain weight unintentionally? | 2.3; 2.6 |
| 1. Did you experience one or more changes in your sensory perception? | 2.6; 2.7 |
| 1. Did you feel dizzy/lightheaded/unsteady? | 2.9; 3.0 |
| 1. Did your skin feel itchy? | 2.3; 2.4 |
| 1. Men only: Do you suffer from an erectile dysfunction? (inability to achieve or to maintain an erection during sexual activity) | N/A; 2.8 |
| 1. Was your sleep impaired? (difficulties in falling asleep, staying asleep or waking up) | 3.0; 3.0 |
| 1. Did you have difficulties in concentrating on things? | 3.3; 3.2 |
| 1. Did you feel depressed? | 3.3; 3.3 |
| 1. Did you worry? | 3.1; 2.9 |
| 1. Did you feel tense and/or irritable? | 2.9; 3.2 |
| 1. Were you limited in doing either your work or other daily activities? | 3.2; 2.8 |
| 1. Were you limited in pursuing your hobbies or other leisure time activities? | 3.1; 2.6 |
| 1. Do you have any trouble doing strenuous activities, like carrying a heavy shopping bag or a suitcase? | 3.3; 2.9 |
| 1. Do you have any trouble taking a long walk? | 3.0; 2.4 |
| 1. Do you need to stay in bed or a chair during the day? | 2.4; 2.4 |

Table 16 mean ratings of questionnaire items by the entire panel: 1st Delphi round

| Questionnaire items from the 1^st^ Delphi round | Overall rating (mean) |
| --- | --- |
|  | AA; PNH |
| 1. In general, do you feel tired? | 3.7; 3.8 |
| 1. Do you have an increased bleeding tendency? | 3.2; 2.7 |
| 1. Have you been in pain? | 2.9; 3.5 |
| 1. Did you experience shortness of breath? | 3.3; 3.3 |
| 1. Did you have a cough? | 2.4; 2.2 |
| 1. Did you have digestive/gastrointestinal problems? | 2.4; 2.9 |
| 1. Did you have difficulties in swallowing things? | N/A; 2.7 |
| 1. Have you noticed changes in hair, skin and/or mucous membranes? | 2.9; 2.4 |
| 1. Did you have swelling/edema of your limbs? | 2.4; 2.1 |
| 1. Have you noticed a dark discoloration of the urine? | N/A; 3.5 |
| 1. Have you noticed a yellowish discoloration of your ‘white of the eye’? | 2.2; 2.9 |
| 1. Did you have fever (from 38.0°C)? | 3.0; 3.1 |
| 1. Did you record a high blood pressure? (upper value >140 mmHg, lower value >80 mmHg) | 3.0; 2.7 |
| 1. Did you record a too low or to high pulse? (< 60 beats/minute or >90 beats/minute) | 2.8; 2.4 |
| 1. Did you experience palpitations? (unpleasant sensation of irregular and/or forceful beating of the heart) | 2.3; 2.6 |
| 1. Did you lose or gain weight unintentionally? | 2.3; 2.5 |
| 1. Did you experience one or more changes in your sensory perception? | 2.5; 2.6 |
| 1. Did you feel dizzy/lightheaded/unsteady? | 2.8; 2.9 |
| 1. Did your skin feel itchy? | 2.2; 2.2 |
| 1. Men only: Do you suffer from erectile dysfunction? (inability to achieve or to maintain an erection during sexual activity) | N/A; 2.9 |
| 1. Was your sleep impaired? (difficulties in falling asleep, staying asleep or waking up) | 2.9; 2.8 |
| 1. Did you have difficulties in concentrating on things? | 3.1; 2.9 |
| 1. Did you feel depressed? | 3.2; 3.2 |
| 1. Did you worry? | 3.0; 2.8 |
| 1. Did you feel tense and/or irritable? | 2.7; 2.9 |
| 1. Were you limited in doing either your work or other daily activities? | 3.2; 3.1 |
| 1. Were you limited in pursuing your hobbies or other leisure time activities? | 3.1; 2.7 |
| 1. Do you have any trouble doing strenuous activities, like carrying a heavy shopping bag or a suitcase? | 3.0; 2.8 |
| 1. Do you have any trouble taking a long walk? | 3.0; 2.6 |
| 1. Do you need to stay in bed or a chair during the day? | 2.5; 2.5 |

Table 17 comparison of AA questionnaire items rating: patients vs. experts

| PRO-AA-Questionnaire items from the 1^st^ Delphi round | Mean rating |
| --- | --- |
|  | Patients; experts |
| 1. In general, do you feel tired? | 3.6; 3.7 |
| 1. Do you have an increased bleeding tendency? | 3.0; 3.6 |
| 1. Have you been in pain? | 2.9; 3.0 |
| 1. Did you experience shortness of breath? | 3.3; 3.2 |
| 1. Did you have a cough? | 2.4; 2.4 |
| 1. Did you have digestive/gastrointestinal problems? | 2.6; 2.1 |
| 1. Have you noticed changes in hair, skin and/or mucous membranes? | 2.8; 3.0 |
| 1. Did you have swelling/edema of your limbs? | 2.4; 2.4 |
| 1. Have you noticed a yellowish discoloration of your ‘white of the eye’? | 2.2; 2.1 |
| 1. Did you have fever (from 38.0°C)? | 2.6; 3.9 |
| 1. Did you record a high blood pressure? (upper value >140 mmHg, lower value >90 mmHg) | 2.8; 3.3 |
| 1. Did you record a too low or to high pulse? (< 60 beats/minute or >90 beats/minute) | 2.9; 2.6 |
| 1. Did you experience palpitations? (unpleasant sensation of irregular and/or forceful beating of the heart) | 2.4; 2.1 |
| 1. Did you lose or gain weight unintentionally?^2^ | 2.3; 2.6 |
| 1. Did you experience one or more changes in your sensory perception? | 2.6; 2.3 |
| 1. Did you feel dizzy/lightheaded/unsteady? | 2.9; 2.7 |
| 1. Did your skin feel itchy? | 2.3; 2.0 |
| 1. Was your sleep impaired? (difficulties in falling asleep, staying asleep or waking up) | 3.0; 2.7 |
| 1. Did you have difficulties in concentrating on things? | 3.3; 2.7 |
| 1. Did you feel depressed? | 3.3; 3.0 |
| 1. Did you worry? | 3.1; 2.9 |
| 1. Did you feel tense and/or irritable? | 2.9; 2.2 |
| 1. Were you limited in doing either your work or other daily activities? | 3.2; 3.3. |
| 1. Were you limited in pursuing your hobbies or other leisure time activities? | 3.1; 3.0 |
| 1. Do you have any trouble doing strenuous activities, like carrying a heavy shopping bag or a suitcase? | 3.3; 2.3 |
| 1. Do you have any trouble taking a long walk? | 3.0; 2.8 |
| 1. Do you need to stay in bed or a chair during the day? | 2.4; 2.6 |

Table 18 comparison of PNH questionnaire items rating: patients vs. experts

| PRO-PNH-Questionnaire items from the 1^st^ Delphi round | Mean rating |
| --- | --- |
|  | Patients; experts |
| 1. In general, do you feel tired? | 3.9; 3.6 |
| 1. Do you have an increased bleeding tendency? | 2.4; 3.3 |
| 1. Have you been in pain? | 3.4; 3.7 |
| 1. Did you experience shortness of breath? | 3.2; 3.5 |
| 1. Did you have a cough? | 2.3; 2.1 |
| 1. Did you have digestive/gastrointestinal problems? | 2.9; 2.9 |
| 1. Did you have difficulties in swallowing things? | 2.5; 3.0 |
| 1. Have you noticed changes in hair, skin and/or mucous membranes? | 2.3; 2.6 |
| 1. Did you have swelling/edema of your limbs? | 1.9; 2.3 |
| 1. Have you noticed a dark discoloration of the urine? | 3.5; 3.3 |
| 1. Have you noticed a yellowish discoloration of your ‘white of the eye’? | 2.8; 3.0 |
| 1. Did you have fever (from 38.0°C)? | 2.7; 3.6 |
| 1. Did you record a high blood pressure  (upper value >140mmHg, lower value >90mmHg) | 2.5; 3.0 |
| 1. Did you record a too low or to high pulse? (< 60 beats/minute or >90 beats/minute) | 2.5; 2.3 |
| 1. Did you experience palpitations?  (unpleasant sensation of irregular and/or forceful beating of the heart) | 2.7; 2.4 |
| 1. Did you lose or gain weight unintentionally? | 2.6; 2.3 |
| 1. Did you experience one or more changes in your sensory perception? | 2.7; 2.4 |
| 1. Did you feel dizzy/lightheaded/unsteady? | 3.0; 2.9 |
| 1. Did your skin feel itchy? | 2.4; 2.0 |
| 1. Men only: Do you suffer from erectile dysfunction? (inability to achieve or to maintain an erection during sexual activity) | 2.8; 3.0 |
| 1. Was your sleep impaired (difficulties in falling asleep, staying asleep or waking up)? | 3.0; 2.6 |
| 1. Did you have difficulties in concentrating on things? | 3.2; 2.6 |
| 1. Did you feel depressed? | 3.3; 3.1 |
| 1. Did you worry? | 2.9; 2.7 |
| 1. Did you feel tense and/or irritable? | 3.2; 2.4 |
| 1. Were you limited in doing either your work or other daily activities? | 2.8; 3.4 |
| 1. Were you limited in pursuing your hobbies or other leisure time activities? | 2.6; 2.9 |
| 1. Do you have any trouble doing strenuous activities, like carrying a heavy shopping bag or a suitcase? | 2.9; 2.7 |
| 1. Do you have any trouble taking a long walk? | 2.4; 2.8 |
| 1. Do you need to stay in bed or a chair during the day? | 2.4; 2.7 |

Table 19 mean ratings of questionnaire items: 2nd Delphi round

| Questionnaire items from the 2^nd^ Delphi round | Overall rating (mean) |
| --- | --- |
|  | AA; PNH |
| 1. In general, do you feel tired? | 3.4; 3.6 |
| 1. Did you experience shortness of breath? | 3.1; 3.2 |
| 1. Do you have an increased bleeding tendency? | 3.0; 2.5 |
| 1. Were you limited in doing either your work or other daily as well as leisure time activities?^#^ | 3.1; 2.8 |
| 1. Did you have difficulties in concentrating on things? | 2.5; 2.7 |
| 1. Do you have any trouble doing strenuous and/or long-lasting activities?^#^ (e.g. carrying a heavy bag, taking long walks) | 3.1; 2.6 |
| 1. Was your mood impaired?^#^ (feeling depressed, being worried, feeling tense and others) | 2.6, 2.8 |
| 1. Did you have fever? (from 38.1°C at least 2 times or once ≥38.3°C) | 2.5; 2.8 |
| 1. Did you record a high blood pressure? (upper value >140mmHg, lower value >90mmHg) | 2.2; 2.0 |
| 1. Was your sleep impaired? (difficulties in falling asleep, staying asleep or waking up) | 2.6; 2.5 |
| 1. Have you been in pain? | 2.5; 3.2 |
| 1. Have you noticed changes in hair, skin and/or mucous membranes? | 2.7; 2.1 |
| 1. Did you feel dizzy/lightheaded/unsteady? | 2.5; 2.5 |
| 1. Did you record a too low or to high pulse? (< 60 beats/minute or >90 beats/minute) | 2.6; 1.8 |
| 1. Did you experience one or more changes in your sensory perception? | 2.1; 2.0 |
| 1. Did you suffer from muscle cramps/spasms? | 2.7; N/A |
| 1. Did you experience tremor^a^ and/or ataxia^b^? (^a^uncontrolled shaking movements of the whole or parts of the body ^b^lack of coordination of muscle movements) | 2.0; N/A |
| 1. Was the time with your family and/or your availability to your children impaired? | 2.5; N/A |
| 1. Have you noticed a dark discoloration of the urine? | N/A; 3.3 |
| 1. Do you need to stay in bed or a chair during the day? | 2.0; 1.9 |
| 1. Did you have digestive/gastrointestinal problems? | 2.2; 2.5 |
| 1. Did you have a cough? | 2.0; 2.2 |
| 1. Did you have swelling/edema of your limbs? | 2.0; 2.2 |
| 1. Did you lose or gain weight unintentionally? | 2.0; 2.4 |
| 1. Did you experience palpitations? (unpleasant sensation of irregular and/or forceful beating of the heart) | 1.7; 2.3 |
| 1. Have you noticed a yellowish discoloration of your ‘white of the eye’? | 1.9; 2.3 |
| 1. Did your skin feel itchy? | 1.7; 1.7 |
| 1. Men only: Do you suffer from erectile dysfunction? (inability to achieve or to maintain an erection during sexual activity) | N/A; 2.2 |
| 1. Did you have difficulties in swallowing things? | N/A; 2.1 |

^#^According to feedbacks from the 1^st^ questionnaire, the question has been merged/combined from several questions due to the similarity of the questions. I.e. “Did you feel depressed?” and “Did you worry?” and “Did you feel tense and/or irritable” to “Was your mood impaired?”. “Were you limited in doing either your work or other daily activities?” and “Were you limited in pursuing your hobbies or other leisure time activities?” to “Were you limited in doing either your work or other daily as well as leisure time activities?”. “Do you have any trouble doing strenuous activities, like carrying a heavy shopping bag or a suitcase?” and “Do you have any trouble taking a long walk?” to “Do you have any trouble doing strenuous and/or long-lasting activities? (e.g. carrying a heavy bag, taking long walks)”.

Table 20 patients' choice from the 3rd Delphi round

| Questionnaire items 3^rd^ Delphi-round | Number of patients votings for inclusion  (AA n = 9, PNH n = 4) |
| --- | --- |
|  | AA; PNH |
| 1. Fatigue, tiredness or a lack of energy | 9; 4 |
| 1. Increased bleeding tendency | 7; 2 |
| 1. Impaired activities of daily living | 7; 3 |
| 1. Dyspnea | 8; 3 |
| 1. Increased bleeding tendency | 7; 2 |
| 1. Muscle cramps | 5; N/A |
| 1. Skin or mucosal changes | 2; 0 |
| 1. Pulse | 5; 2 |
| 1. Insomnia | 1; 1 |
| 1. Depression, sad | 4; 2 |
| 1. Impaired family time | 3; N/A |
| 1. Dizziness | 4; 1 |
| 1. Pain | 2; 4 |
| 1. Fever | 3; 1 |
| 1. Concentration difficulties | 8; 4 |
| 1. Gastro-intestinal problems | 5; 3 |
| 1. Bloodpressure | 5; 1 |
| 1. Disturbed sensory perception | 3; 0 |
| 1. Weight loss or weight gain | 1; 0 |
| 1. Peripheral edema | 1; 1 |
| 1. Cough | 0; 0 |
| 1. Bed or chair ridden | 0; 0 |
| 1. Tremor or ataxia | 3; N/A |
| 1. Jaundice | 2; 0 |
| 1. Itching | 1; 3 |
| 1. Palpitations | 4; 3 |
| 1. Hemoglobinuria | N/A; 3 |
| 1. Strenous activities | 6; 1 |
| 1. Dysphagia | N/A; 2 |
| 1. Erectile dysfunction | N/A; 0 |

N/A: not applicable

**Decisions on modifications of the questionnaire by the expert panel (3^rd^ Delphi round)**

Table 21 experts' modification of the questionnaire

| 1) Symptoms related to hematopoietic stem cell transplantation (‘*changes in hair, skin and/or mucous membranes*’ associated to GvHD) were excluded due to the very different symptom course after transplantation and little specificity of these symptoms for AA/PNH. |
| --- |
| 2) The items ‘*impairment in performing strenuous activities*’; ‘*impairment in activities of daily living*’ and ‘*impaired family time*’ were combined with the item ‘*fatigue*’ resulting in a single question covering the general performance in daily life. |
| 3) ‘*Palpitation*’ was included in the AA-PRO-questionnaire since atrial fibrillation is an important complication in the elderly, in particular with anemia (Xu et al. American Journal of Cardiology, 2015), and there is data suggesting that AA patients could have an elevated risk (Hu et al. Circulation Journal, 2018). |
| 4) The item ‘*concentration problems*’ was supplemented with memory dysfunction on the basis of personal experience. |
| 5) Although most AA patients rated ‘*tremor and ataxia*’ as not relevant, the experts determined to include these items together with ‘*neuropathic symptoms’* as well-known adverse effects of ciclosporin. |
| 6) ‘*Insomnia*’ was not considered to be specifically related to AA or PNH and was excluded. |
| 7) Although not rated amongst the highest in the second consensus round, the items ‘*dysphagia*’; ‘*jaundice*’ and ‘*erectile dysfunction*’ were assessed as being particularly relevant as a marker for hemolysis in the course of PNH (DeZern et al. Hematology/Oncology Clinics, 2015). |

**Provisional PRO-AA and PRO-PNH questionnaire (expert centered)**

Table 22 provisional PRO-AA/PNH questionnaire according to experts’ choice

| 1. Symptom Term: Fatigue | | | | | | | |
| --- | --- | --- | --- | --- | --- | --- | --- |
| Did you feel fatigue, tiredness or a lack of energy? | | | | | | | |
| ☐ Yes | | | | ☐ No | | | |
| What was the severity of your fatigue, tiredness or lack of energy at its worst? | | | | | | | |
| ☐ mild | | ☐ moderate | | ☐ severe | | ☐ very severe | |
| How much did fatigue, tiredness or lack of energy interfere with your usual or daily activities? | | | | | | | |
| ☐ not at all | ☐ a little bit | | ☐ somewhat | | ☐ quite a bit | | ☐ very much |

| 1. Symptom Term: Fever | | | | | | | | | | | | | |
| --- | --- | --- | --- | --- | --- | --- | --- | --- | --- | --- | --- | --- | --- |
| Did you have fever (once >38.5°C or twice >38.0°C lasting at least 1 hour, measured at the ear)? | | | | | | | | | | | | | |
| Yes | | | | | | | No | | | | | | |
| 1. **Symptom Term: Bleeding** | | | | | | | | | | | | | |
| Did you experience bleeding (for females: includes increased menstrual bleeding, not normal-strong/long bleeding)? | | | | | | | | | | | | | |
| Yes | | | | | | | No | | | | | | |
| Where was the bleeding? | | | | | | | | | | | | | |
| Nose | Mouth | | | Skin and/or soft tissue (e.g. bruises, petechiae) | | gastrointestinal (e.g. blood mixed with stool, blood in vomit) | | in urine | | vaginal | | | during coughing |
| - 1. **Symptom Term: Nose bleeding** | | | | | | | | | | | | | |
| How severe was your nose bleeding at its worst? | | | | | | | | | | | | | |
| less than 30 minutes during 24 hours | | | | | | | | | | | | | |
| more than 30 minutes during 24 hours | | | | | | | | | | | | | |
| so strong that red cell transfusion was necessary (over routine transfusion needs) | | | | | | | | | | | | | |
| so strong, that your blood pressure decreased severely (systolic fall of at least 50 mmHg) and red blood cell transfusion was necessary (over routine transfusion needs) | | | | | | | | | | | | | |
| - 1. Symptom Term: Oral bleeding | | | | | | | | | | | | | |
| How severe was your oral bleeding (from the mouth) at its worst? | | | | | | | | | | | | | |
| less than 30 minutes during 24 hours | | | | | | | | | | | | | |
| more than 30 minutes during 24 hours | | | | | | | | | | | | | |
| so strong that red cell transfusion was necessary (over routine transfusion needs) | | | | | | | | | | | | | |
| so strong, that your blood pressure decreased severely (systolic fall of at least 50 mmHg) and red blood cell transfusion was necessary (over routine transfusion needs) | | | | | | | | | | | | | |
| - 1. **Symptom Term: Skin and soft tissue bleeding** | | | | | | | | | | | | | |
| How severe was your skin and/or soft tissue bleeding at its worst? | | | | | | | | | | | | | |
| occasionally punctiform hemorrhages (less than 2.5 cm in extent)  and/or spontaneously superficial bruise(s) | | | | | | | | | | | | | |
| spread punctiform haemorrhages (over 2.5 cm in extent) and/or deep  skin/soft tissue bleeding | | | | | | | | | | | | | |
| so severe, that red cell transfusion was necessary (over routine transfusion needs) | | | | | | | | | | | | | |
| so severe, that your blood pressure decreased severely (systolic fall of at least 50 mmHg) and red blood cell transfusion was necessary (over routine transfusion needs) | | | | | | | | | | | | | |
| - 1. **Symptom Term: Gastrointestinal bleeding** | | | | | | | | | | | | | |
| How severe was your gastrointestinal bleeding at its worst? | | | | | | | | | | | | | |
| occasionally punctiform hemorrhages (less than 2.5 cm in extent)  and/or spontaneously superficial bruise(s) | | | | | | | | | | | | | |
| spread punctiform hemorrhages (over 2.5 cm in extent) and/or deep  skin/soft tissue bleeding | | | | | | | | | | | | | |
| so severe, that red cell transfusion was necessary (over routine transfusion needs) | | | | | | | | | | | | | |
| so severe, that your blood pressure decreased severely (systolic fall of at least 50 mmHg) and red blood cell transfusion was necessary (over routine transfusion needs) | | | | | | | | | | | | | |
| - 1. **Symptom Term: Blood in urine** | | | | | | | | | | | | | |
| How severe was your bleeding in the urine at its worst? | | | | | | | | | | | | | |
| blood was not visible in urine, but detectable by laboratory test | | | | | | | | | | | | | |
| visible blood in Urine (salmon colored, reddish, brownish) | | | | | | | | | | | | | |
| so severe, that red cell transfusion was necessary (over routine transfusion needs) | | | | | | | | | | | | | |
| so severe, that your blood pressure decreased severely (systolic fall of at least 50 mmHg) and red blood cell transfusion was necessary (over routine transfusion needs) | | | | | | | | | | | | | |
| - 1. **Symptom Term: Vaginal bleeding** | | | | | | | | | | | | | |
| Are you pregnant, or is there a possibility for pregnancy? | | | | | | | | | | | | | |
| Yes | | | | | | | No | | | | | | |
| How severe was your vaginal bleeding at its worst (refers to unexpected bleeding out of the normal cycle or bleeding heavier than normal or breakthrough bleeding)? | | | | | | | | | | | | | |
| bleeding in form of spotting | | | | | | | | | | | | | |
| bleeding more than spotting | | | | | | | | | | | | | |
| so severe, that red cell transfusion was necessary (over routine transfusion needs) | | | | | | | | | | | | | |
| so severe, that your blood pressure decreased severely (systolic fall of at least 50 mmHg) and red blood cell transfusion was necessary (over routine transfusion needs) | | | | | | | | | | | | | |
| 1. **Symptom Term: Shortness of breath** | | | | | | | | | | | | | |
| Did you feel short of breath? | | | | | | | | | | | | | |
| Yes | | | | | | | No | | | | | | |
| What was the severity of your shortness of breath at its worst? | | | | | | | | | | | | | |
| mild | | | moderate | | | | severe | | | | very severe | | |
| How much did shortness of breath interfere with your usual or daily activities? | | | | | | | | | | | | | |
| not at all | | a little bit | | | somewhat | | | | quite a bit | | | very much | |
| 1. **Symptom Term: Pain** | | | | | | | | | | | | | |
| Did you have pain? | | | | | | | | | | | | | |
| Yes | | | | | | | No | | | | | | |
| Where did you have pain (multiple answers possible) | | | | | | | | | | | | | |
| chest | | abdominal | | | head | | | | muscle/joint | | | *Open input box* | |
| How often did you have pain? | | | | | | | | | | | | | |
| rarely | | | occasionally | | | | frequently | | | | almost constantly | | |
| What was the severity of your pain at its worst? | | | | | | | | | | | | | |
| mild | | | moderate | | | | severe | | | | very severe | | |
| How much did pain interfere with your usual or daily activities? | | | | | | | | | | | | | |
| not at all | | | a little bit | | | | somewhat | | | | quite a bit | | |
| 1. **Symptom Term: Mood** | | | | | | | | | | | | | |
| Have you often been bothered by feeling down, depressed or hopeless? | | | | | | | | | | | | | |
| Yes | | | | | | | No | | | | | | |
| What was the severity of these feelings at its worst? | | | | | | | | | | | | | |
| mild | | | moderate | | | | severe | | | | very severe | | |
| How much did these feelings interfere with your usual or daily activities? | | | | | | | | | | | | | |
| not at all | | a little bit | | | somewhat | | | | quite a bit | | | very much | |
| 1. **Symptom Term: Memory and concentration** | | | | | | | | | | | | | |
| Did you have problems with memory and/or concentration? | | | | | | | | | | | | | |
| Yes | | | | | | | No | | | | | | |
| What was the severity of your problems with memory and/or concentration at its worst? | | | | | | | | | | | | | |
| mild | | | moderate | | | | severe | | | | very severe | | |
| How much did problems with memory and/or concentration interfere with your usual or daily activities? | | | | | | | | | | | | | |
| not at all | | a little bit | | | somewhat | | | | quite a bit | | | very much | |
| 1. **Symptom Term: Palpitations (only in PRO-AA questionnaire))** | | | | | | | | | | | | | |
| Did you have a pounding or racing heartbeat (palpitations)? | | | | | | | | | | | | | |
| Yes | | | | | | | No | | | | | | |
| What was the severity of your pounding or racing heartbeat (palpitations) at its worst? | | | | | | | | | | | | | |
| mild | | | moderate | | | | severe | | | | very severe | | |
| How often did you feel a pounding or racing heartbeat (palpitations)? | | | | | | | | | | | | | |
| not at all | | a little bit | | | somewhat | | | | quite a bit | | | very much | |
| 1. **Symptom Term: Tremor (only in PRO-AA questionnaire)** | | | | | | | | | | | | | |
| Did you experience uncontrolled shaking movements (tremor) of individual body parts (e.g. hands)? | | | | | | | | | | | | | |
| Yes | | | | | | | No | | | | | | |
| What was the severity of the uncontrolled shaking movements (tremor) at its worst? | | | | | | | | | | | | | |
| mild | | | moderate | | | | severe | | | | very severe | | |
| How much did the uncontrolled shaking movements (tremors) interfere with your usual or daily activities? | | | | | | | | | | | | | |
| not at all | | a little bit | | | somewhat | | | | quite a bit | | | very much | |
| 1. **Symptom Term: Muscle cramps (only in PRO-AA questionnaire)** | | | | | | | | | | | | | |
| Did you experience muscle cramps (e.g. calf)? | | | | | | | | | | | | | |
| Yes | | | | | | | No | | | | | | |
| What was the severity of your muscle cramps at its worst? | | | | | | | | | | | | | |
| mild | | | moderate | | | | severe | | | | very severe | | |
| How much did the muscle cramps interfere with your usual or daily activities? | | | | | | | | | | | | | |
| not at all | | a little bit | | | somewhat | | | | quite a bit | | | very much | |
| 1. **Symptom Term: Numbness and tingling (only in PRO-AA questionnaire)** | | | | | | | | | | | | | |
| Did you experience numbness or tingling in your hands or feet? | | | | | | | | | | | | | |
| Yes | | | | | | | No | | | | | | |
| What was the severity of your numbness and tingling at its worst? | | | | | | | | | | | | | |
| mild | | | moderate | | | | severe | | | | very severe | | |
| How much did numbness and tingling interfere with your usual or daily activities? | | | | | | | | | | | | | |
| not at all | | a little bit | | | somewhat | | | | quite a bit | | | very much | |
| 1. **Symptom Term: Dark urine [hemoglobinuria] (only in PRO-PNH questionnaire)** | | | | | | | | | | | | | |
| Did you have dark (reddish or cola-colored) urine (in particular in the morning) | | | | | | | | | | | | | |
| Yes | | | | | | | No | | | | | | |
| 1. **Symptom Term: jaundice (only in PRO-PNH questionnaire)** | | | | | | | | | | | | | |
| Was your skin and/or whites of the eyes yellowish colored (jaundice)? | | | | | | | | | | | | | |
| Yes | | | | | | | No | | | | | | |
| What was the severity of the yellowish coloring of your skin and/or whites  of your eyes? | | | | | | | | | | | | | |
| mild | | | moderate | | | | severe | | | | very severe | | |
| 1. **Symptom Term: Difficulty swallowing [dysphagia] (only in PRO-PNH questionnaire)** | | | | | | | | | | | | | |
| Did you have difficulties with swallowing? | | | | | | | | | | | | | |
| Yes | | | | | | | No | | | | | | |
| What was the severity of your difficulties with swallowing at its worst? | | | | | | | | | | | | | |
| mild | | | moderate | | | | severe | | | | very severe | | |
| Did you have to adjust your eating habits due to the difficulties with swallowing? | | | | | | | | | | | | | |
| never | | rarely | | | occasionally | | | | frequently | | | almost constantly | |
| 1. **Symptom Term: Erectile dysfunction (only in PRO-PNH questionnaire)** | | | | | | | | | | | | | |
| Did you have difficulties getting or keeping an erection? | | | | | | | | | | | | | |
| Yes | | | No | | | | Not sexually active | | | | Prefer not to answer | | |
| What was the severity of your difficulty getting or keeping an erection its worst? | | | | | | | | | | | | | |
| mild | | | moderate | | | | severe | | | | very severe | | |
| 1. **Open Question** | | | | | | | | | | | | | |
| Did you have any other symptoms/problems, which were not queried above? | | | | | | | | | | | | | |
| Yes | | | | | | | No | | | | | | |
| What kind of symptoms/problems did you have? | | | | | | | | | | | | | |
| [Open input box] | | | | | | | | | | | | | |

**Provisional PRO-AA and PRO-PNH questionnaire (patient centered)**

Table 23 provisional patient centered PRO-AA/PNH questionnaire

| 1. Symptom Term: Fatigue | | | | | | | |
| --- | --- | --- | --- | --- | --- | --- | --- |
| Did you feel fatigue, tiredness or a lack of energy? | | | | | | | |
| ☐ Yes | | | | ☐ No | | | |
| What was the severity of your fatigue, tiredness or lack of energy at its worst? | | | | | | | |
| ☐ mild | | ☐ moderate | | ☐ severe | | ☐ very severe | |
| How much did fatigue, tiredness or lack of energy interfere with your usual or daily activities? | | | | | | | |
| ☐ not at all | ☐ a little bit | | ☐ somewhat | | ☐ quite a bit | | ☐ very much |

| 1. Symptom Term: Bleeding | | | | | | | |
| --- | --- | --- | --- | --- | --- | --- | --- |
| Did you experience bleeding (for females: includes increased menstrual bleeding, not normal-strong/long bleeding)? | | | | | | | |
| Yes | | | | No | | | |
| Where was the bleeding? | | | | | | | |
| Nose  Mouth  Skin and/or soft tissue (e.g. bruises, petechiae)  gastrointestinal (e.g. blood mixed with stool, blood in vomit)  in urine  vaginal  during coughing | | | | | | | |
| - 1. **Symptom Term: Nose bleeding** | | | | | | | |
| How severe was your nose bleeding at its worst? | | | | | | | |
| less than 30 minutes during 24 hours | | | | | | | |
| more than 30 minutes during 24 hours | | | | | | | |
| so strong that red cell transfusion was necessary (over routine transfusion needs) | | | | | | | |
| so strong, that your blood pressure decreased severely (systolic fall of at least 50 mmHg) and red blood cell transfusion was necessary (over routine transfusion needs) | | | | | | | |
| - 1. Symptom Term: Oral bleeding | | | | | | | |
| How severe was your oral bleeding (from the mouth) at its worst? | | | | | | | |
| less than 30 minutes during 24 hours | | | | | | | |
| more than 30 minutes during 24 hours | | | | | | | |
| so strong that red cell transfusion was necessary (over routine transfusion needs) | | | | | | | |
| so strong, that your blood pressure decreased severely (systolic fall of at least 50 mmHg) and red blood cell transfusion was necessary (over routine transfusion needs) | | | | | | | |
| - 1. **Symptom Term: Skin and soft tissue bleeding** | | | | | | | |
| How severe was your skin and/or soft tissue bleeding at its worst? | | | | | | | |
| occasionally punctiform hemorrhages (less than 2.5 cm in extent)  and/or spontaneously superficial bruise(s) | | | | | | | |
| spread punctiform hemorrhages (over 2.5 cm in extent) and/or deep  skin/soft tissue bleeding | | | | | | | |
| so severe, that red cell transfusion was necessary (over routine transfusion needs) | | | | | | | |
| so severe, that your blood pressure decreased severely (systolic fall of at least 50 mmHg) and red blood cell transfusion was necessary (over routine transfusion needs) | | | | | | | |
| - 1. **Symptom Term: Gastrointestinal bleeding** | | | | | | | |
| How severe was your gastrointestinal bleeding at its worst? | | | | | | | |
| occasionally punctiform hemorrhages (less than 2.5 cm in extent)  and/or spontaneously superficial bruise(s) | | | | | | | |
| spread punctiform hemorrhages (over 2.5 cm in extent) and/or deep  skin/soft tissue bleeding | | | | | | | |
| so severe, that red cell transfusion was necessary (over routine transfusion needs) | | | | | | | |
| so severe, that your blood pressure decreased severely (systolic fall of at least 50 mmHg) and red blood cell transfusion was necessary (over routine transfusion needs) | | | | | | | |
| - 1. **Symptom Term: Blood in urine** | | | | | | | |
| How severe was your bleeding in the urine at its worst? | | | | | | | |
| blood was not visible in urine, but detectable by laboratory test | | | | | | | |
| visible blood in Urine (salmon colored, reddish, brownish) | | | | | | | |
| so severe, that red cell transfusion was necessary (over routine transfusion needs) | | | | | | | |
| so severe, that your blood pressure decreased severely (systolic fall of at least 50 mmHg) and red blood cell transfusion was necessary (over routine transfusion needs) | | | | | | | |
| - 1. **Symptom Term: Vaginal bleeding** | | | | | | | |
| Are you pregnant, or is there a possibility for pregnancy? | | | | | | | |
| Yes | | | | No | | | |
| How severe was your vaginal bleeding at its worst (refers to unexpected bleeding out of the normal cycle or bleeding heavier than normal or breakthrough bleeding)? | | | | | | | |
| bleeding in form of spotting | | | | | | | |
| bleeding more than spotting | | | | | | | |
| so severe, that red cell transfusion was necessary (over routine transfusion needs) | | | | | | | |
| so severe, that your blood pressure decreased severely (systolic fall of at least 50 mmHg) and red blood cell transfusion was necessary (over routine transfusion needs) | | | | | | | |
| 1. **Symptom Term: Shortness of breath** | | | | | | | |
| Did you feel short of breath? | | | | | | | |
| Yes | | | | No | | | |
| What was the severity of your shortness of breath at its worst? | | | | | | | |
| mild | | moderate | | severe | | very severe | |
| How much did shortness of breath interfere with your usual or daily activities? | | | | | | | |
| not at all | a little bit | | somewhat | | quite a bit | | very much |
| 1. **Symptom Term: Mood** | | | | | | | |
| Have you often been bothered by feeling down, depressed or hopeless? | | | | | | | |
| Yes | | | | No | | | |
| What was the severity of these feelings at its worst? | | | | | | | |
| mild | | moderate | | severe | | very severe | |
| How much did these feelings interfere with your usual or daily activities? | | | | | | | |
| not at all | a little bit | | somewhat | | quite a bit | | very much |
| 1. **Symptom Term: Memory and concentration** | | | | | | | |
| Did you have problems with memory and/or concentration? | | | | | | | |
| Yes | | | | No | | | |
| What was the severity of your problems with memory and/or concentration at its worst? | | | | | | | |
| mild | | moderate | | severe | | very severe | |
| How much did problems with memory and/or concentration interfere with your usual or daily activities? | | | | | | | |
| not at all | a little bit | | somewhat | | quite a bit | | very much |
| 1. **Symptom Term: Palpitations** | | | | | | | |
| Did you have a pounding or racing heartbeat (palpitations)? | | | | | | | |
| Yes | | | | No | | | |
| What was the severity of your pounding or racing heartbeat (palpitations) at its worst? | | | | | | | |
| mild | | moderate | | severe | | very severe | |
| How often did you feel a pounding or racing heartbeat (palpitations)? | | | | | | | |
| not at all | a little bit | | somewhat | | quite a bit | | very much |
| 1. **Symptom Term: Muscle cramps (only in PRO-AA questionnaire)** | | | | | | | |
| Did you experience muscle cramps (e.g. calf)? | | | | | | | |
| Yes | | | | No | | | |
| What was the severity of your muscle cramps at its worst? | | | | | | | |
| mild | | moderate | | severe | | very severe | |
| How much did the muscle cramps interfere with your usual or daily activities? | | | | | | | |
| not at all | a little bit | | somewhat | | quite a bit | | very much |
| 1. **Symptom Term: Dizziness (only in PRO-AA questionnaire)** | | | | | | | |
| Did you feel dizzy? | | | | | | | |
| Yes | | | | No | | | |
| What was the severity of your dizziness at its worst? | | | | | | | |
| mild | | moderate | | severe | | very severe | |
| How much did dizziness interfere with your usual or daily activities? | | | | | | | |
| not at all | a little bit | | somewhat | | quite a bit | | very much |
| 1. **Symptom Term: Trouble doing strenuous activities (only in PRO-AA questionnaire)** | | | | | | | |
| Did you have any trouble doing strenuous activities, like carrying a heavy bag or a suitcase? | | | | | | | |
| not at all | | a little | | quite a bit | | very much | |
| How much did your trouble doing strenuous activities interfere with your usual or daily activities? | | | | | | | |
| not at all | a little bit | | somewhat | | quite a bit | | very much |
| 1. **Symptom Term: Gastro-intestinal problems** | | | | | | | |
| Did you have gastro-intestinal problems? | | | | | | | |
| Yes | | | | No | | | |
| What kind of gastro-intestinal problem did you have? | | | | | | | |
| nausea  vomiting  heartburn  diarrhea (loos or watery stools)  constipation  bloating  increased passing of gas | | | | | | | |
| - 1. **Symptom Term: Nausea** | | | | | | | |
| How often did you have nausea? | | | | | | | |
| rarely | | occasionally | | frequently | | almost constantly | |
| What was the severity of your nausea at its worst? | | | | | | | |
| mild | | moderate | | severe | | very severe | |
| How much did your nausea interfere with your usual or daily activities? | | | | | | | |
| not at all | a little bit | | somewhat | | quite a bit | | very much |
| - 1. **Symptom Term: Vomiting** | | | | | | | |
| How often did you have vomiting? | | | | | | | |
| rarely | | occasionally | | frequently | | almost constantly | |
| What was the severity of your vomiting at its worst? | | | | | | | |
| mild | | moderate | | severe | | very severe | |
| How much did your vomiting interfere with your usual or daily activities? | | | | | | | |
| not at all | a little bit | | somewhat | | quite a bit | | very much |
| - 1. **Symptom Term: Heartburn** | | | | | | | |
| How often did you have heartburn? | | | | | | | |
| rarely | | occasionally | | frequently | | almost constantly | |
| What was the severity of your heartburn at its worst? | | | | | | | |
| mild | | moderate | | severe | | very severe | |
| How much did your heartburn interfere with your usual or daily activities? | | | | | | | |
| not at all | a little bit | | somewhat | | quite a bit | | very much |
| - 1. **Symptom Term: Diarrhea** | | | | | | | |
| How often did you have loose or watery stools (diarrhea)? | | | | | | | |
| rarely | | occasionally | | frequently | | almost constantly | |
| How much did your loose or watery stools (diarrhea) interfere with your usual or daily activities? | | | | | | | |
| not at all | a little bit | | somewhat | | quite a bit | | very much |
| - 1. **Symptom Term: Constipation** | | | | | | | |
| What was the severity of your constipation at its worst? | | | | | | | |
| mild | | moderate | | severe | | very severe | |
| How much did your constipation interfere with your usual or daily activities? | | | | | | | |
| not at all | a little bit | | somewhat | | quite a bit | | very much |
| - 1. **Symptom Term: Bloating** | | | | | | | |
| How often did you have bloating of the abdomen (belly)? | | | | | | | |
| rarely | | occasionally | | frequently | | almost constantly | |
| What was the severity of your bloating of the abdomen (belly) at its worst? | | | | | | | |
| mild | | moderate | | severe | | very severe | |
| How much did your bloating interfere with your usual or daily activities? | | | | | | | |
| not at all | a little bit | | somewhat | | quite a bit | | very much |
| 1. **Symptom Term: Pain (only in PRO-PNH questionnaire)** | | | | | | | |
| Did you have pain? | | | | | | | |
| Yes | | | | Yes | | | |
| Where did you have pain (multiple answers possible) | | | | | | | |
| chest | abdominal | | head | | muscle/joint | | *Open input box* |
| How often did you have pain? | | | | | | | |
| rarely | | occasionally | | frequently | | almost constantly | |
| What was the severity of your pain at its worst? | | | | | | | |
| mild | | moderate | | severe | | very severe | |
| How much did pain interfere with your usual or daily activities? | | | | | | | |
| not at all | | a little bit | | somewhat | | quite a bit | |
| 1. **Symptom Term: Dark urine [hemoglobinuria] (only in PRO-PNH questionnaire)** | | | | | | | |
| Did you have dark (reddish or cola-colored) urine (in particular in the morning) | | | | | | | |
| Yes | | | | No | | | |
| 1. **Symptom Term: Difficulty swallowing [dysphagia] (only in PRO-PNH questionnaire)** | | | | | | | |
| Did you have difficulties with swallowing? | | | | | | | |
| Yes | | | | Yes | | | |
| What was the severity of your difficulties with swallowing at its worst? | | | | | | | |
| mild | | mild | | mild | | mild | |
| Did you have to adjust your eating habits due to the difficulties with swallowing? | | | | | | | |
| never | never | | never | | never | | never |
| 1. **Symptom Term: Itching (only in PRO-PNH questionnaire)** | | | | | | | |
| Did you have an itchy skin? | | | | | | | |
| Yes | | | | No | | | |
| What was the severity of your itchy skin at its worst? | | | | | | | |
| mild | | moderate | | severe | | very severe | |
| How much did your itchy skin interfere with your usual or daily activities? | | | | | | | |
| not at all | a little bit | | somewhat | | quite a bit | | very much |
| 1. **Open Question** | | | | | | | |
| Did you have any other symptoms/problems, which were not queried above? | | | | | | | |
| Yes | | | | No | | | |
| What kind of symptoms/problems did you have? | | | | | | | |
| [Open input box] | | | | | | | |
